# Metagenomic discovery of microbial eukaryotes in stool microbiomes

**DOI:** 10.1128/mbio.02063-24

**Published:** 2024-08-29

**Authors:** Audra L. Crouch, Laine Monsey, Molly Rambeau, Cameron Ramos, Joseph M. Yracheta, Matthew Z. Anderson

**Affiliations:** 1 Department of Microbiology, The Ohio State University, Columbus, Ohio, USA; 2 Native BioData Consortium, Eagle Butte, South Dakota, USA; 3 Department of Microbial Infection and Immunity, The Ohio State University, Columbus, Ohio, USA; 4 Center for Genomic Science Innovation, University of Wisconsin - Madison, Madison, Wisconsin, USA; 5 Laboratory of Genetics, University of Wisconsin - Madison, Madison, Wisconsin, USA; University of Michigan-Ann Arbor, Ann Arbor, Michigan, USA

**Keywords:** eukaryome, fungi, protozoa, DNA extraction, metagenomes

## Abstract

Host-associated microbiota form complex microbial communities that are increasingly associated with host behavior and disease. While these microbes include bacterial, archaeal, viral, and eukaryotic constituents, most studies have focused on bacteria due to their dominance in the human host and available tools for investigation. Accumulating evidence suggests microbial eukaryotes in the microbiome play pivotal roles in host health, but our understandings of these interactions are limited to a few readily identifiable taxa because of technical limitations in unbiased eukaryote exploration. Here, we combined cell sorting, optimized eukaryotic cell lysis, and shotgun sequencing to accelerate metagenomic discovery and analysis of host-associated microbial eukaryotes. Using synthetic communities with a 1% microbial eukaryote representation, the eukaryote-optimized cell lysis and DNA recovery method alone yielded a 38-fold increase in eukaryotic DNA. Automated sorting of eukaryotic cells from stool samples of healthy adults increased the number of microbial eukaryote reads in metagenomic pools by up to 28-fold compared to commercial kits. Read frequencies for identified fungi increased by 10,000× on average compared to the Human Microbiome Project and allowed for the identification of novel taxa, *de novo* assembly of contigs from previously unknown microbial eukaryotes, and gene prediction from recovered genomic segments. These advances pave the way for the unbiased inclusion of microbial eukaryotes in deciphering determinants of health and disease in the host-associated microbiome.

**IMPORTANCE:**

Microbial eukaryotes are common constituents of the human gut where they can contribute to local ecology and host health, but they are often overlooked in microbiome studies. The lack of attention is due to current technical limitations that are heavily biased or poorly recovered DNA from microbial eukaryotes. We developed a method to increase the representation of these eukaryotes in metagenomic sequencing of microbiome samples that allows to improve their detection compared to prior methods and allows for the identification of new species. Application of the technique to gut microbiome samples improved detection of fungi, protists, and helminths. New eukaryotic taxa and their encoded genes could be identified by sequencing a small number of samples. This approach can improve the inclusion of eukaryotes into microbiome research.

## INTRODUCTION

Microbial communities are globally ubiquitous entities composed of bacteria, archaea, viruses, and eukaryotes that shape the biotic and abiotic makeup of the Earth and its macroscopic life. Animal and plant systems have evolved alongside colonizing microbes to produce complex cross-kingdom interactions (1–3). The human gut remains one of the most well-characterized host-associated niches, where more than a decade of research has defined the bacterial and viral repertoires and their contributions to microbial ecology, host physiology, host development, and the balance between health and disease (4–14). In stark contrast, little attention has been directed to the role of resident gut microbial eukaryotes, “the eukaryome,” to human health and disease, which is further crippled by an incomplete definition of resident taxa (15–19).

Fungal, protist, and helminth species contribute to core functions in the human gut microbiome through host and microbe interactions (20–22). Fungal species of *Candida* can modulate microbiota directly through the secretion of signaling molecules that inhibit the growth of specific bacterial taxa (23, 24). Furthermore, microbial eukaryotes shape bacterial composition and organization by spatially guiding community assemblage or favoring taxa *via* metabolic interactions (25–34). Shaping community composition is facilitated by the large size of microbial eukaryotes compared to their bacterial counterparts, which typically differ by an order of magnitude. Eukaryotes can also interact with the host immune system to indirectly alter the microbiota (17, 18, 35). For example, secretion of the immunoregulatory glycoprotein ES-62 by the nematode *Acanthocheilonema vitae* suppresses interleukin (IL)-17-mediated inflammation and inhibits the overgrowth of commensal bacteria including Firmicutes and Proteobacteria (36, 37). The resulting microbiome assemblage can directly contribute to host physiology, ranging from social behaviors to autoimmune disease and tumors (16–19, 38, 39), and is often mediated through bidirectional communication with host immunity (17, 36, 40–42). However, the key taxa that drive these responses are often unknown or obscured by a lack of taxonomical resolution (32, 43–45).

Major technical and methodological limitations hinder the study of microbial eukaryote composition and abundance. First, the relatively lower abundance of eukaryotes in the microbiome has produced a reliance on amplicon-based sequencing of the 18S ribosomal RNA gene and associated internal transcribed spacer regions (e.g., ITS1 and ITS2) for taxonomical classification and abundance measurements in the vast majority of studies (46, 47). Amplicon-based ITS profiling suffers from amplification biases, limited ability to detect non-fungal organisms, copy number variation between strains and species, and reliance on curated databases of previously described taxa that restricts discovery (48–53). Consequently, analysis of the same human stool sample by the two ITS primer sets could result in substantially different estimates of fungal taxa (48, 54). Second, ITS amplicons systematically exclude protists and helminths despite extensive historical evidence for their presence (48, 55–57). Third, metagenomic approaches to the human gut microbiome suffer from insufficient sequence capture of microbial eukaryotes. On average, only ~0.01% of metagenomic reads map to eukaryotic microbes across human metagenomic studies, with some variation based on body site, health status, geography, and collection method (48, 58, 59). Taken together, amplicon-based sequencing leaves substantial gaps in definitions of the eukaryome, while metagenomics efforts that do not use an “ultra-deep” method produce insufficient information to reconstruct the community assembly of microbial eukaryotes in healthy adult populations. Resolving deficits in metagenomic approaches would facilitate the construction of more complete definitions of the microbiome and the contributions of microbial eukaryotes.

Here, we developed and tested a eukaryotic enrichment approach that allows for metagenomic sequencing of these microbes in complex communities, including human fecal samples. Physical separation of microbial eukaryotes from the majority population of bacteria and archaea proved to be a crucial step in the success of this approach in human fecal samples that were aided by the optimization of cell lysis and DNA isolation steps. Together, this approach vastly outperformed commercial microbiome kits and allows for the identification of all captured microbial eukaryotes, eukaryotic metagenome-assembled genome (MAG) construction, and insight into the gene content of microbial eukaryotes from the gut to expand our understanding of microbial roles in metabolism and human disease.

## RESULTS

### Mechanical lysis improves DNA extraction from microbial eukaryotes

**Fig 1:**
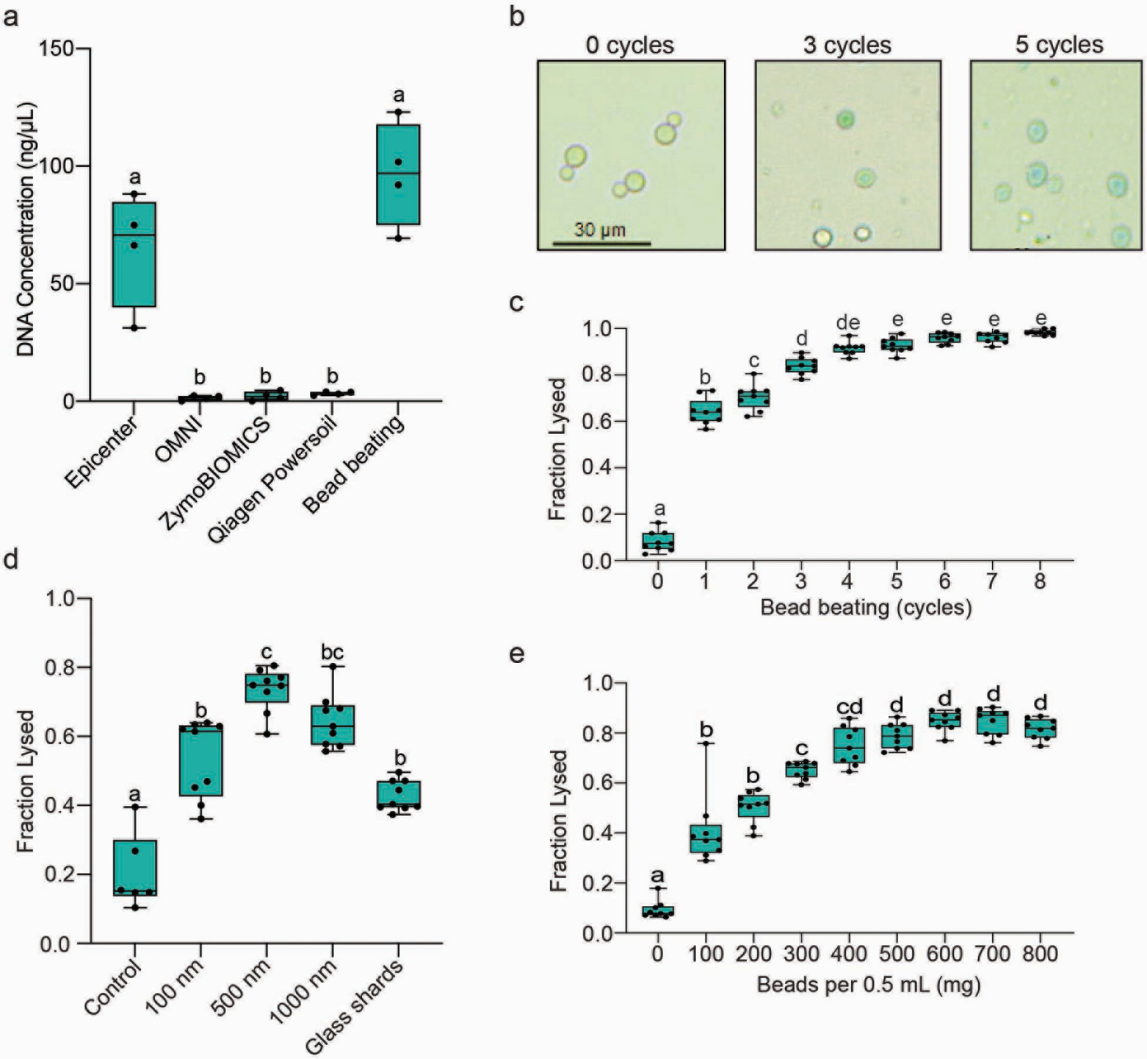
Optimization of DNA recovery from fungal cells in microbiome samples. (a) Synthetic stool (500 mg) was seeded with 1 × 10^8^ *C. neoformans* cells, and the efficiency of five different DNA recovery approaches for microbiome analysis was tested (*N* = 4). (b) *C. neoformans* cells were bead-beaten with 100 μm beads for different numbers of cycles (1 min on, 2 min off) and incubated with 0.4% trypan blue solution. Intact cells appear clear, while lysed cells stain blue. Images were taken at 40× magnification. Scale bar = 30 μm. The fraction of *C. neoformans* cells lysed, as indicated by trypan blue staining, was measured across a range of bead-beating cycles with 100 μm glass beads (c), different glass bead sizes and glass shards for 5 cycles (d), and varying masses of 100 μm glass beads per 0.5 mL of sample for five cycles (e). Six fields of view were analyzed per replicate. *N* = 9, 9, and 5 for each respective panel. Boxplots mark the interquartile ranges with the median marked and whiskers extending to the outermost data points. Letters denote statistically significant differences in cell lysis fractions based on a one-way ANOVA and Tukey’s post hoc test at *P* < 0.05.

Failure to efficiently recover DNA from microbial eukaryotes present in microbiome samples likely contributes to existing gaps in taxonomic characterization (48). To determine the efficacy of recovering eukaryotic DNA from stool samples by available approaches, microbe-free synthetic stool was seeded with 1.0 × 10^8^ cells per milliliter (mL) of the fungus *Cryptococcus neoformans*, which have a tightly crosslinked cell wall and thick capsule of exo-glycoprotein that makes it a particularly recalcitrant organism to DNA extractions (60). DNA recovery was performed from equal aliquots of synthetic stool seeded with *C. neoformans* using three common microbiome preparation kits, a fungal-specific DNA isolation kit, and mechanical lysis *via* bead beating. Either bead beating or the fungal-specific kit efficiently recovered DNA from *C. neoformans*, whereas all three microbiome kits had significantly reduced DNA yields (Fig. 1a, one-way ANOVA (F(4,15) = 36.0, *P* < 1.0E-4). This highlights a potential major limitation to microbial eukaryote identification using standard microbiome approaches and identified mechanical disruption *via* bead beating as a viable alternative to kits based on its improved performance and ability to be modified.

To optimize DNA release during bead beating, we incrementally adjusted individual parameters in bead-beating protocols and assessed their effect on maximizing cell lysis. The azo dye trypan blue was added to all optimization trials to distinguish between lysed cells, which appear blue, and intact cells that lack staining (Fig. 1b). First, the effect of increasing the number of bead-beating cycles (1 minute on, 2 minutes off) on cell lysis was assessed. Cell lysis increased with added cycles, reaching peak effectiveness beginning at five cycles (Fig. 1b and c). Next, beads of diverse sizes ranging from 100 to 1,000 nanometers (nm) were tested alongside glass shards and no beads, which served as a negative control. Beads averaging 500 nm in diameter proved most effective at lysing *C. neoformans* cells, a prototypical yeast cell of ~5 microns in diameter, compared to other bead sizes and glass shards (Fig. 1d). Finally, the number of beads per sample was assayed in 100 mg increments. Cell lysis increased with added beads up to 600 mg and decreased slightly with greater amounts (Fig. 1e). Thus, lysis of a durable representative yeast was most effective using 600 mg of 500 nm glass beads for a duration of five cycles of bead beating.

Buffers used to homogenize stool samples can profoundly affect cell lysis and DNA recovery (61, 62). To independently test buffer composition on cell lysis, we extracted DNA from *C. neoformans* cells by bead beating in buffers from a representative microbiome extraction kit, the commonly used extraction buffer cetyltrimethylammonium bromide (CTAB), and a buffered detergent solution without 3% sodium dodecyl sulfate (SDS; TENT) or including the detergent (TENTS). Surprisingly, CTAB and the kit-associated buffer performed poorly compared to TENT and TENTS buffers during bead beating (Fig. S1a). Unexpectedly, quantification of DNA yields from *C. neoformans* in synthetic stool did not initially correlate with an increasing number of seeded cells, suggesting carryover of contaminating materials from the synthetic stool inhibited accurate quantification of recovered DNA. However, the addition of a phenol-chloroform extraction, secondary chloroform extraction, and magnetic bead purification removed the contaminating substance(s) and led to a strong association between the seeded number of cells and DNA recovery (cor = 0.984, Pearson’s test = 10.99, df = 4, *P*-value = 1.00E-3, Fig. S1b).

The cell lysis and DNA recovery approach were extended to five different microbial eukaryotes to assess its broader applicability. Extraction efficiency was quantified for single-species populations of fungi: *Aspergillus fumigatus* hyphae, *A. fumigatus* spores, *Candida albicans*, *Pichia pastoris*, *Saccharomyces cerevisiae*, and the protist *Leishmania major*. The developed approach performed best for the yeast species *P. pastoris and S. cerevisiae* with extraction efficiencies of 64.6% and 68.9%, respectively (Fig. S2). Reduced efficiency of DNA extraction from *C. albicans* (18.2%) may be attributable to relatively higher chitin and β-glucans content and few mannoproteins in its cell wall compared to other ascomycetes (63). DNA extraction of the protozoan species *L. major* also showed moderate success with an extraction efficiency of 30.8% (Fig. S2). Hyphae and spores of *A. fumigatus* tested cell morphologies not present in many yeasts but that could be present in fungi. DNA recovery from *A. fumigatus* spores was quite poor (5.4%), whereas hyphae readily released DNA (58.5%). Thus, successful and consistent DNA extractions can be obtained from eukaryotic cells with different structures and a range of morphologies.

### Eukaryotic DNA is enriched from a synthetic microbial community

**Fig 2:**
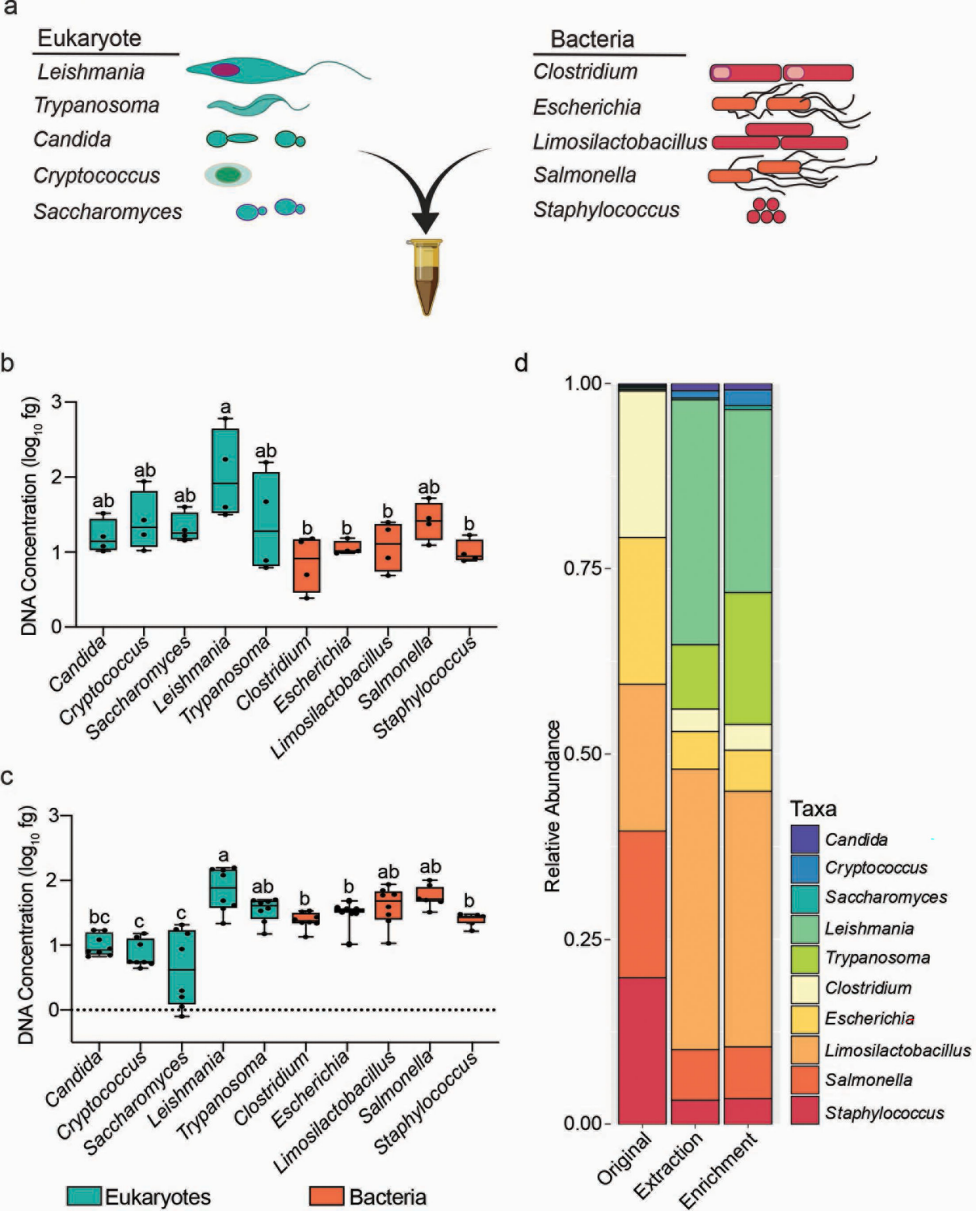
Enrichment of microbial eukaryote DNA from synthetic communities. (a). Five bacterial and five eukaryotic species were grown separately and seeded in 500 mg of synthetic stool as either equivalent (1:1, 10^7^ cells/mL per species) or bacterial-biased (99:1, 10^7^ eukaryotic cells/mL and 10^9^ bacterial cells/mL) ratios with equal representation of species within the bacterial or eukaryotic groups. The developed eukaryome protocol was performed on either 1:1 (b) or 99:1 (c) microbial communities and the fraction of DNA recovered from each species was plotted. Recovered DNA was determined using quantitative PCR with species-specific primers against a single copy genomic locus specific to each organism and quantified using a standard curve of known DNA concentrations. Boxplots mark the interquartile ranges with the median marked and whiskers extending to the outermost data points. Letters indicate statistically different groups based on a one-way ANOVA and Tukey’s post hoc test at *P* < 0.05. *N* = 4 for 1:1 communities and *N* = 8 for 99:1 communities. (d). The relative abundance of DNA for each species is given for the 99:1 bacterial biased initial community (left), following DNA extraction (middle), or after enrichment by DpnI digestion (right). Each species is color-coded as indicated.

To test the efficacy of the developed approach to recover microbial eukaryotes in mixed microbial populations, we built a 10-member synthetic community consisting of five eukaryotes (*C. albicans*, *C. neoformans*, *S. cerevisiae*, *L. major*, and *Trypanosoma brucei*) and five bacterial species (*Clostridioides difficile*, *Escherichia coli*, *Limosilactobacillus reuteri*, *Salmonella typhimurium*, and *Staphylococcus aureus*) (Fig. 2a). Species for the mock community were seeded in synthetic stool at either equivalent ratios (1:1) or the 99:1 (bacteria:eukaryote) ratio estimated in the human gut. We extracted DNA from the mock community using the developed approach and quantified DNA recovery from each species by quantitative PCR (qPCR) with species-specific primers built against single-copy loci (Table S1). Processing the 1:1 synthetic community recovered similar quantities of DNA from each species, although there was a bias for recovering *L. major* DNA (one-way ANOVA, F(9,30) = 3.078, *P* = 0.0098, Fig. 2b). For the 99:1 bacteria-dominated mock community, bacterial DNA should be recovered in a near 100-fold excess of eukaryotic DNA. By contrast, the cell lysis and DNA recovery process enriched for eukaryotic DNA in the synthetic population (one-way ANOVA, F(9, 70) =17.47, *P* < 0.0001, Fig. 2c). DNA from the protists *L. major* and *T. brucei* was present in equal concentration to the seeded bacteria, and the fungal eukaryotes (*C. albicans, C. neoformans*, and *S. cerevisiae*) were present at only a 10-fold reduction compared to bacterial species. Collectively, optimization for eukaryotic DNA recovery alone produced a 38× enrichment of eukaryotic DNA compared to their initial cellular representation in the defined community (Fig. 2d). Thus, the cell lysis and DNA recovery approach selectively enrich microbial eukaryotes in synthetic communities.

We attempted to further improve eukaryotic DNA representation in the synthetic community by exploiting common differences in base methylation of DNA between bacteria and eukaryotes. Incubating DNA from the gram-negative bacterium *E. coli* and/or *C. neoformans* with the DpnI restriction endonuclease, which cleaves 5′-G^m6^ATC-3′ commonly found in bacterial genomes, either alone or mixed in equivalent ratios degraded *E. coli* DNA but did not alter the size distribution of *C. neoformans* DNA (Fig. S3a and b). Purification of DpnI-treated DNA with magnetic beads recovered *C. neoformans* but not *E. coli* DNA from digested pools (Fig. S2c). However, treatment of DNA harvested from the 99:1 bacteria-dominated samples with DpnI had only a minimal impact on further eukaryotic enrichment (43-fold vs 38-fold without DpnI-treatment) from synthetic communities despite effectively digesting DNA from each bacterial species when incubated alone (Fig. 2d; Fig. S3d).

### Microbial eukaryotes can be selectively sorted from stool

**Fig 3:**
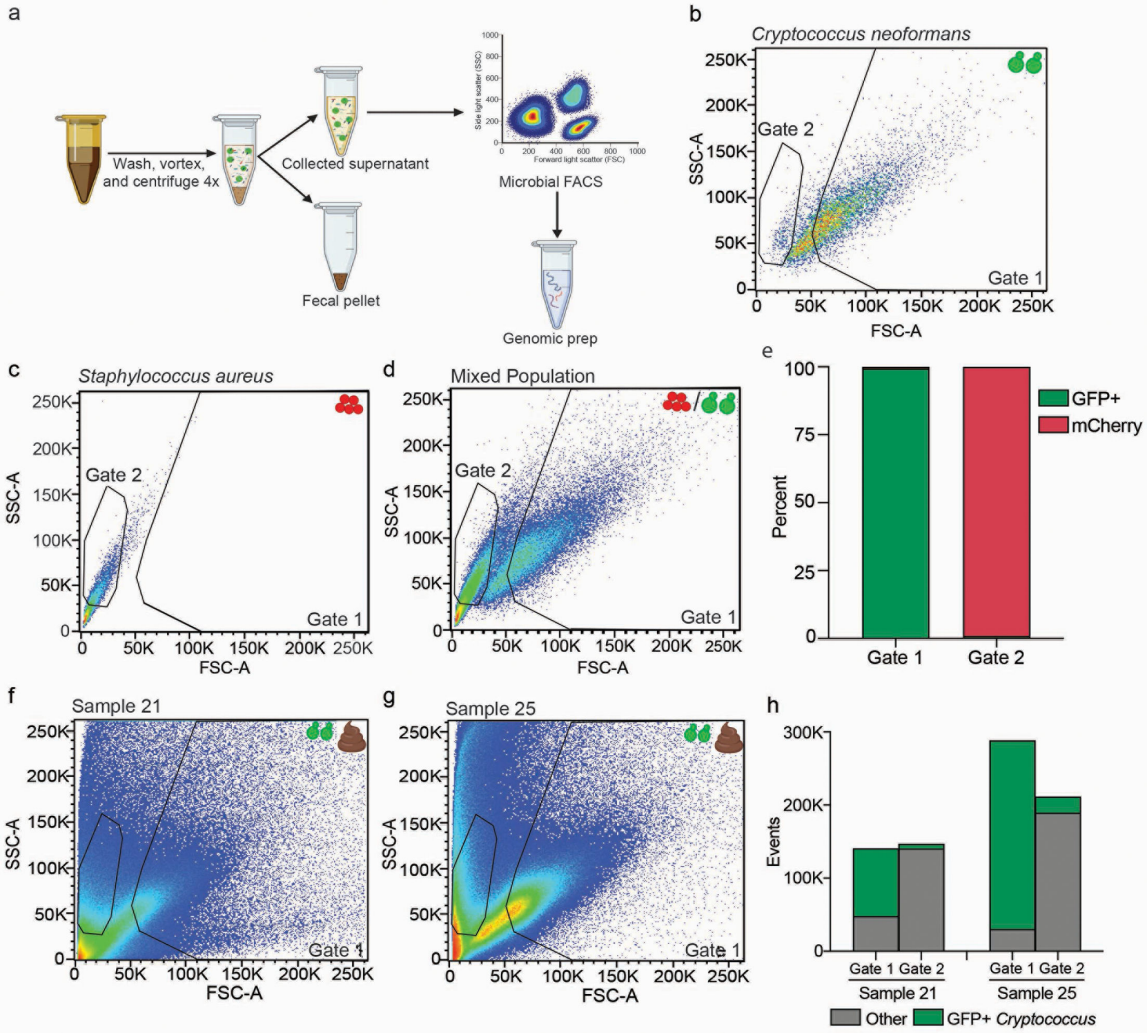
Isolation of microbial eukaryotes by cell sorting. (a). Schematic for microbial isolation and fluorescence-activated cell sorting (FACS) of microbial eukaryotes from stool. Gates were built that distinguish either GFP^+^ *C. neoformans* (Gate 1) or mCherry^+^ *S. aureus* (Gate 2) based on forward and side scatter either individually (b, c) or mixed in an equivalent 50:50 mixture (d). (e) The fraction of GFP and mCherry events captured in Gates 1 and 2 from mixing cells was quantified. The stool was spiked with GFP^+^ *C. neoformans* and cells isolated by FACS using the previously established Gate 1 and Gate 2 for two fecal samples, sample 21 (f) and sample 25 (g). (h) The number of events captured in Gates 1 and 2 for two fecal samples and the proportion of GFP^+^ *C. neoformans* cells or other events in each group are indicated in green and gray, respectively.

The expectation of relatively low microbial eukaryote abundances in host-associated microbiomes strongly argued for the inclusion of additional processing steps to enrich these populations in the stool. A differential centrifugation and cell sorting approach were developed to physically separate microbial eukaryotes from the bacterial population (Fig. 3a). We spiked human fecal samples with GFP^+^ *C. neoformans*, which are not found in the human gut, to monitor the retention of microbial eukaryotes during sample processing *via* fluorescence. Testing a range of centrifugation conditions on spiked fecal samples found speeds between 300 and 400 ×*g* for 3 minutes pelleted the fecal matter while retaining GFP^+^ *C. neoformans* in the supernatant (Fig. S4a and b). Attempts to extract additional GFP^+^ *C. neoformans* from pelleted fecal material by repeated resuspension, washing, and centrifugation produced a decay curve in fluorescence signal, indicating that *C. neoformans* was being partitioned to the supernatant. Alternatively, undisturbed settling of resuspended fecal matter by gravity over 10 minutes was equally effective to centrifugation in separating GFP^+^ *C. neoformans* from fecal material (Fig. S4c). Sonication of the sample prior to centrifugation did not increase *C. neoformans* yields, suggesting minimal adherence to fecal material (Fig. S4d). Similarly, GFP^+^ *P. pastoris*, mCherry^+^ *L. major*, *C. albicans*, and *Caenorhabditis elegans* eggs could be recovered under these conditions when seeded individually into stool samples (Fig. S4e through h).

Further enrichment of microbial eukaryotes from complex populations was possible by sorting cells based on differences in cell size, shape, and intracellular contents. Gates to sort cells by fluorescence-activated cell sorting (FACS) were constructed using GFP^+^ *C. neoformans* and mCherry-containing *S. aureus* to mark the representative position of eukaryotic and bacterial cells, respectively (Fig. 3b and c). When mixed in equal numbers, *C. neoformans* and *S. aureus* cells formed two distinct populations that could each be recovered with little carryover (Fig. 3d). The fungal gate (Gate 1) contained 99.35% GFP^+^ *C. neoformans* cells, while the bacterial gate (Gate 2) included only 0.86% *C*. *neoformans* cells (Fig. 3e). The frequency of events in Gate 1 for nine other eukaryote populations (*A. fumigatus* hyphae, *A. fumigatus* spores, *C. albicans* hyphae, *C. albicans* yeast, *P. pastoris*, *S. cerevisiae*, *L. major*, *T. brucei*, and *C. elegans* eggs and adults) was enriched (range 4.8%–88.6%, median 56%) compared to *S. aureus*, *C. difficile*, or *S. typhimurium* (present at <1% in Gate 1, Fig. S5). While Gate 1 was extremely successful in capturing fungi, sorted protist and helminth samples were present in Gates 1 and 2 at roughly equal frequencies. Importantly, most of the *C. difficile* and the *S. typhimurium* populations fell outside of both gates in the extreme corner of the plot, representative of a much smaller cell size. Thus, Gate 1 is highly selective for fungal species and some protists and helminths, which can also be captured by Gate 2.

To further support the defined gates in eukaryotic enrichment, 10^7^ eukaryotes (*C. albicans*, *L. major*, or *P. pastoris*) were mixed with 10^9^ *S. typhimurium,* and the frequency of each species in Gate 1 and Gate 2 was determined by flow cytometry. As expected from sorting pure cultures, Gate 1 strongly selected for the fungal cells (785× and 168,000× for *C. albicans* and *P. pastoris* compared to *S. typhimurium*, respectively), and Gate 2 enriched for the protist *L. major* (800× compared to *S. typhimurium*, Fig. S6) in each mixed population.

To test the developed sorting strategy for the enrichment of microbial eukaryotes from human fecal samples, 10^7^ GFP^+^ *C. neoformans* cells were spiked into 200 mg of stool from two donors, the microbial contents were separated by centrifugation, and the microbes in the eukaryotic and bacterial gates recovered by FACS (Fig. 3f and g). Gate 1, which was defined by *C. neoformans*, contained a large population of GFP^+^ cells (Fig. 3h; Fig. S7a and c), whereas Gate 2, defined by the mCherry-labeled *S. aureus*, contained few GFP^+^ events (Fig. 3h; Fig. S7b and d). The dramatic increase in GFP^+^ *C. neoformans* in Gate 1 compared to Gate 2 (14.3× and 11.7× enrichment in samples 21 and 25, respectively) indicated that pre-defined gates established by monoculture microbes performed similarly in fecal samples. Importantly, GFP^–^ events captured in Gate 1 indicates resident eukaryotes in the donor stool that were isolated alongside the GFP-labeled *C. neoformans* cells. Taken together, Gate 1 strongly selects fungal cells, and both gates can enrich protists and helminths with species-specific variation in gate performance.

### Eukaryotic enrichment in stool outperforms prior metagenomic efforts

**Fig 4:**
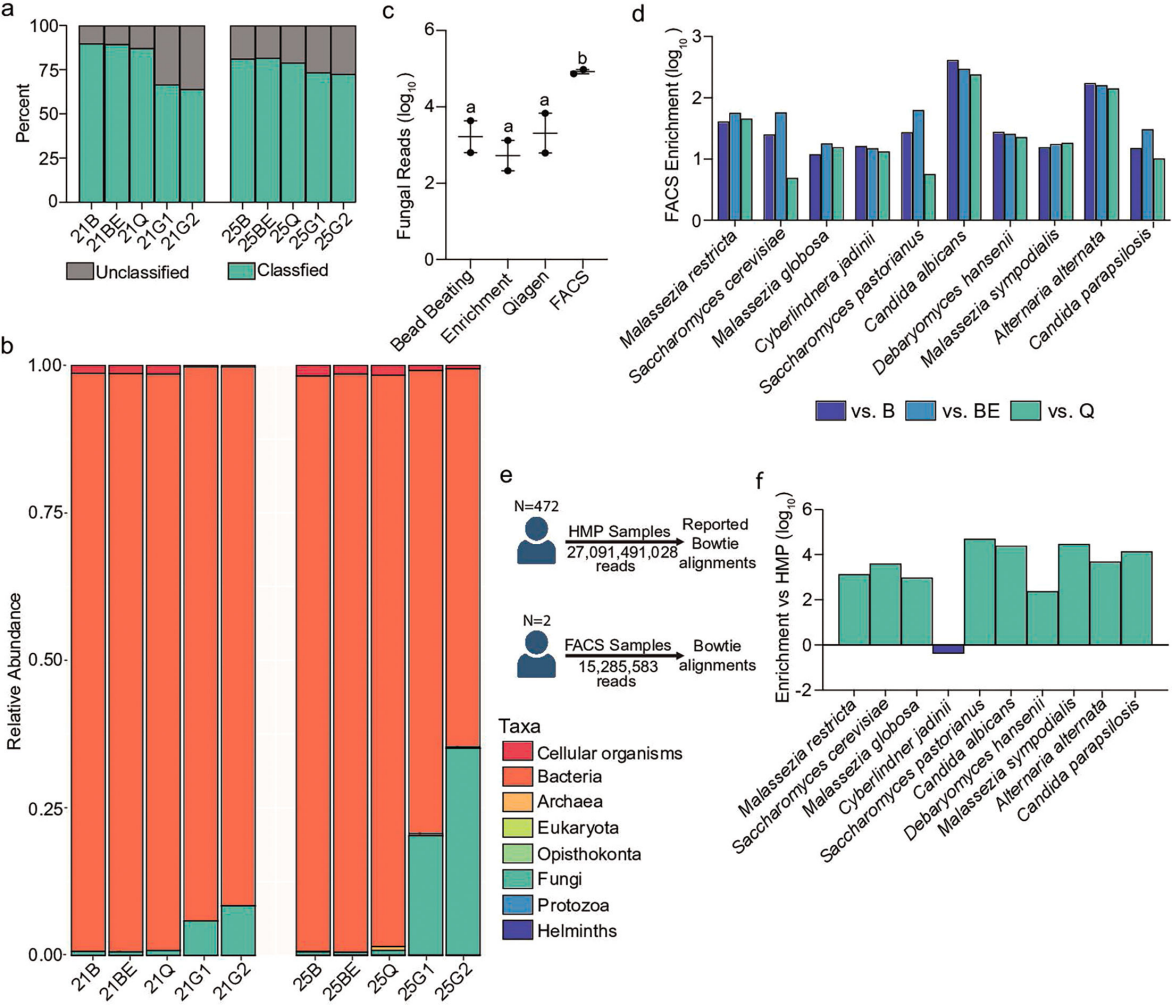
FACS-based eukaryotic enrichment outperforms prior metagenomics efforts. (a) Two fecal samples were extracted and sequenced using metagenomics by one of four approaches, bead beating (B), bead beating and DpnI treatment (BE), Qiagen Powersoil DNA kit (Q), and cell sorting of gates defined in Fig. 1 (G1 or G2). Reads were taxonomically defined by Kraken and categorized as either matching a database compiled from RefSeq (classified) or having no match (unclassified). (b) Classified reads were assigned to major groups of microbial organisms from each of the five groups and plotted as a fraction of the total. (c) The number of fungal reads from each method was quantified for the stool samples after removing all *C. neoformans* reads. *N* = 2. (d) The ratio of reads assigned to the 10 most abundant Human Microbiome Project (HMP) fungal species from the FACS enrichment approach was compared to either bead beating (B), bead beating and DpnI treatment (BE), or Qiagen Powersoil (Q) methods. (e) Schematic of eukaryotic read abundance comparisons with the Human Microbiome Project. (f) The normalized read frequency from the FACS approach and the full HMP metagenomics repository were calculated for each of the 10 most abundant fungal species identified by HMP and plotted as a ratio.

The effectiveness of the combined approach for eukaryotic enrichment by cell sorting and optimized DNA recovery was tested by inoculating 10^7^ GFP^+^ *C. neoformans* cells into 200 mg from each of two human stool samples. Four 200 mg aliquots of *C. neoformans*-seeded stool from each sample were prepared for metagenomic sequencing by one of four different methods: Qiagen DNeasy Powersoil Pro kit with the included bead-beating steps, bead beating as developed using the synthetic community, bead beating with enrichment using DpnI, and FACS-sorted samples followed by bead beating, and sequenced to a depth of 40 million reads (Fig. S8; Table S2). Taxonomy could be assigned to between 64.1% and 89.9% of all reads (Fig. 4a). Interestingly, the percentage of unclassified reads increased substantially in the FACS-sorted pools for both samples (23.6% and 7.70% on average for samples 21 and 25, respectively) compared to DNA extraction without sorting (Student’s *t*-test; Sample 21: *t*(3) = 15.982, *P* = 5.86E-3, Sample 25: *t*(3) = 7.99, *P* = 4.40E-3).

Classified reads showed eukaryotic enrichment in FACS-prepared samples. Bacterial species overwhelmingly dominated samples prepared using the Qiagen approach, with only between 0.68% and 0.73% eukaryotic classified reads in these samples (Fig. 4b; Table S2). The use of bead beating without sorting failed to recapitulate the increased eukaryotic representation found in synthetic communities, containing only 0.54% eukaryotic reads on average, and was not aided by DpnI treatment of sample DNA. By contrast, FACS sorting increased the representation of eukaryotic reads from these two samples. FACS gates in samples 21 and 25 recovered between 3.90%–5.38% and 14.9%–25.5% of reads belonging to microbial eukaryotes, respectively, representing a 7-fold to 28-fold enrichment compared to the Qiagen commercial kit. Fungi compromised 99.5% of assigned eukaryotic reads from FACS-gated DNA pools on average. The *C. neoformans* spike-in allowed us to estimate that between 1 and 5 million microbial eukaryotes were present in the 200 mg of prepared fecal material. When removing reads from spiked-in *C. neoformans* cells from FACS-sorted samples, the abundance of fungal reads remained statistically greater by more than an order of magnitude compared to the other approaches (one-way ANOVA, F(1, 3) =22.24, *P* = 0.0150, Fig. 4c). At a species level, FACS-sorted pools contained between 4.89- and 411-fold enrichment for fungal organisms compared to non-sorted samples with an average of 67.2-fold enrichment across the 10 fungi determined to be most abundant in the human gut from the Human Microbiome Project (HMP; Fig. 4d; Table S3).

FACS-sorted samples were consistently enriched for eukaryotes compared to HMP metagenomic pools. To compare microbial eukaryote read abundances directly with the metagenomic read pool for the Human Microbiome Project, reads from the two FACS gates were pooled across the two samples. This read pool from FACS-sorted microbes was aligned to the reference genomes for the 10 most abundant eukaryotes identified for the HMP cohort as described in Nash et al. (48), normalized to the total read count in each pool, and compared (Fig. 4e). In general, FACS sorting improved recovery of fungal representation by three orders of magnitude compared to HMP metagenomic sequence archives (median = 4,415 fold; range 0.41-fold to 49,886-fold, Fig. 4f). For example, a total of 2,426 *C*. *albicans* reads were detected in 55 of the 472 metagenomic samples from HMP, whereas 34,132 combined *C. albicans* reads were found between the two sequenced samples here (Table S3). Only *Cyberlindnera jadinii* was less abundant in the sorted samples in this study, which may be linked to dietary differences between these cohorts (64).

### Sorting eukaryotes reveals protists in the human gut

Our metagenomics approach identified protists in both stool samples that are often not considered in amplicon-based sequencing of the human microbiome or buried in unenriched metagenomics (47). Thousands of reads from protist genomes were present in both donor samples, approximately half of which corresponded to *Blastocystis* species. The other half of the protist reads corresponded to a diverse set of eukaryotic species commonly found in livestock and meat products and may represent consumed or transient microbes. For example, *Babesia bovis* and *Theileria equi* most likely are transient species resulting from interactions with large animals, tick vectors, or diet (65–67). However, the detection of *Trichomonas tenax* likely represents resident protists colonizing or passing through the human gut and supports its recent identification in another human population by metagenomic sequencing (68).

### Inference of eukaryome function from FACS-sorted pools

**Fig 5:**
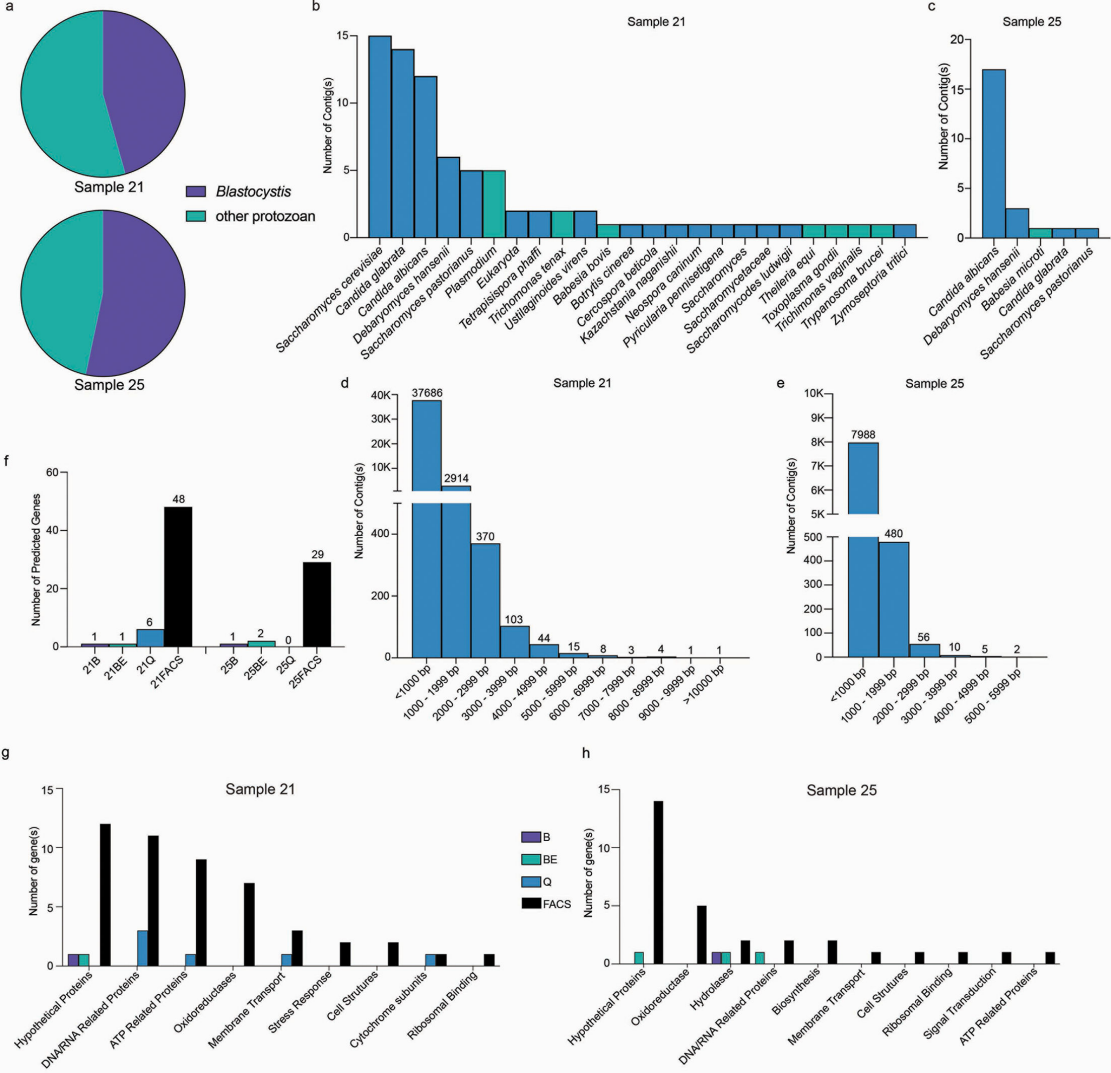
Novel eukaryotic taxa and associated genes are uncovered by metagenomic approaches. (a) The frequency of classified reads assigned to protists by Kraken2 are shown for Blastocystis and all other protists. Contigs greater than 1 Kb assembled for microbial eukaryotes from FACS enriched gates were taxonomically assigned for sample 21 (b) and sample 25 (c). Unclassified reads were assembled into contigs and plotted by size (>1 Kb) for sample 21 (d) and sample 25 (e). (f) Gene prediction in contigs was performed using Augustus and Diamond and quantified for the four metagenomic approaches: bead beating (B), bead beating and DpnI treatment (BE), Qiagen Powersoil DNA kit (Q), and sorted gates together (FACS). Predicted genes from fecal sample 21 (g) and sample 25 (h) were assigned functions using gene ontology (GO).

The application of metagenomic sequencing to eukaryote-enriched samples provides a rich resource for discovering new microbial taxa and functionally informative contigs. The considerable number of recovered *C. neoformans* reads from sorted samples produced eukaryotic contigs up to 12 Kb and demonstrated the feasibility of performing metagenome-assembled genome (MAG) building of eukaryome species (Fig. S9). Following the removal of *C. neoformans* reads, we produced 79 and 23 contigs from FACS-sorted samples 21 and 25 that were greater than 1 kb long, respectively (Fig. 5b and c). *Saccharomyces* and *Candida* species (e.g., *S. cerevisiae, C. glabrata,* and *C. albicans*) comprised the majority of contigs from both samples (Fig. 5b and c). Even though a smaller proportion of eukaryotic reads were obtained for Sample 21, the constructed contigs contained much greater species diversity, including multiple protist species. In contrast to the FACS-sorted pools, contig assembly using the other tested approaches for metagenomic sequencing produced 0–6 contigs. All six recovered contigs from the best-performing alternative sequencing pool (21Q) belonged to *S. cerevisiae*.

We hypothesized that the assembly of the substantial number of unclassified reads from FACS-sorted pools into taxonomically informative contigs may provide additional insight into eukaryotic members of the human gut microbiome. Unclassified FACS-sorted reads for samples 21 and 25 built 41,149 and 8,541 additional contigs, with some extending over 10 Kb (Fig. 5d and e). Many contigs were similar to highly prevalent eukaryotes identified in these samples, including *C. albicans* (95% identity over 315 nucleotides (nt)) and *C. neoformans* (89% identity across 895 nt). Half of the longest contigs built from unclassified reads failed to assign any taxonomic level (e.g., contigs from 5 to 12 Kb, *N* = 34), highlighting the large number of unknown microbes in this pool. However, contigs with taxonomic classification gave unexpected insights into the gut microbiota. For example, sample 21 contained a 6,022 nt contig with strong similarity to *Schistosoma* genomes (81% identity).

To infer the function of constructed contigs, gene prediction was first employed to the assembled sequences obtained from each of the four metagenomics methods. FACS-sorted samples produced contigs with a greater number of predicted eukaryotic genes compared to all other groups (Fig. 5f). Genes in reconstructed contigs from FACS-sorted samples represent a wide spectrum of biological processes. Full-length open reading frames in constructed contigs have predicted functions from nucleic acid regulation to membrane transport and metabolism (Fig. 5g and h). Six functional categories were found in common between the two fecal samples analyzed and may indicate an increased requirement for these genes in gut resident eukaryotes. These jointly held functions center on protein regulation, including metabolism (e.g., hydrolyases, oxidoreductases, and ATP-related proteins). Of note is the high number of hypothetical proteins identified from sorted samples that lack an assigned function and may contribute to eukaryotic functions critical for colonization in this niche.

## DISCUSSION

Low representation in microbiome samples and technical limitations have impeded the investigation of microbial eukaryotes residing within the human gastrointestinal tract. The approach developed here provides a mechanism to expand our understanding of the microbiome through eukaryote enrichment *via* cell sorting and optimized mechanical disruption of recovered cells. Metagenomic sequencing of two sorted stool samples produced rich eukaryotic profiles that outperformed any prior effort in healthy adults. Sequences for known and unknown fungi, protists, and putative helminths were recovered from the human gut. Applying this approach broadly across host niches, conditions, and populations will incorporate the eukaryome into frameworks of human health and disease.

Metagenomic approaches to microbial eukaryote identification in metazoan-associated microbiomes address systematic bias found in amplicon-based community profiling. Over the last decade, profiling methods for bacterial and archaeal communities migrated from amplicon-based approaches to metagenomics after limitations were revealed, including amplification biases, sequencing errors that can misassign taxa, and the inability to access gene content information (69, 70). Our approach removes taxonomic biases present in ITS or 18S amplicon-based sequencing and allows for functional analysis of all recovered eukaryote sequences. The fraction of microbial eukaryote sequences obtained between the human samples differed, but it is unclear how much of this reflects technical or inter-individual variability. Importantly, the sample with fewer eukaryotic reads displayed greater taxonomic diversity, highlighting the significant advantages of this approach regardless of the specific fraction of recovered reads assigned to eukaryotes.

Mechanical lysis to extract DNA from microbial eukaryotes proved to be critical for eukaryotic enrichment. Bead beating has been a long-standing gold standard for cell lysis in fungi and other microbes (71) and proved most effective at breaking open resilient fungal cells and various protozoa. Bead beating was less effective against fungal spores, which likely reflects their highly durable nature and smaller size. Indeed, size is likely a key factor in the selective disruption of microbial eukaryotes using this approach as bead beating under the optimized conditions was less effective for all assayed bacteria (Fig. 2b and c). The DNA extraction methods that significantly improved the recovery of fungal DNA also did not include filtering steps to separate cell debris from nucleic acids, which are common procedures in microbiome kits, and may directly contribute to the unexpectedly low yields of eukaryotic DNA from metagenomics studies (48). Collectively, our data suggest that kits should be avoided or substantially modified when planning to investigate microbial eukaryotes.

Sorting microbes from fecal samples is necessary under these conditions for targeted investigation of microbial eukaryotes. Investment in producing an approach to enrich for microbial eukaryotes from complex communities worked well in synthetic communities seeded in synthetic stool but failed to perform well in human stool. It is possible that eukaryotes are present in such low abundances that the bead-beating approach alone was insufficient to expand their recovery, although the Qiagen Powersoil Kit did recover eukaryote sequences at similar levels to bead beating alone. We postulate that synthetic stool does not fully reproduce the properties of human feces and led to a protocol trained well for synthetic stool but not human feces. The use of microbial cell sorting was a key step in eukaryote enrichment in pools built for metagenomic sequencing. Populations of 100,000 cells were used for each FACS gate to build sequencing libraries but did not require any specialized workflows. Success in sorting cells and obtaining metagenomic libraries in these smaller populations demonstrates the ability to target microbial populations of interest in microbiome research by FACS, including those beyond microbial eukaryotes. Isolating eukaryotic cells was facilitated by their larger size compared to bacteria and archaea. Indeed, isolation and sequencing of the dense population of microbes in the lower left corner found it to be predominantly bacterial, as would be expected given their smaller size (Fig. 3f and g). Thus, sorting microbes from complex communities allows users to select defined regions of cell size and shape distributions to capture different microbial populations. As demonstrated here, the use of monoculture samples and fluorescent microbes can further guide gate refinement to obtain the desired microbial subset.

Metagenomic approaches provide needed insight into defining the full eukaryome. There remains an incomplete consensus on what eukaryotic taxa are resident in the human gut because of taxonomical inconsistencies and a lack of focused attention stemming from methodological concerns (48, 59, 72, 73). Furthermore, the assignment of many gut-resident eukaryotic taxa arose from their associations with virulence during dysbiosis, which downplays the presence of strictly commensal taxa in healthy individuals (e.g., *Alternaria*). Identifying related but genetically distinct contigs to known pathobiont eukaryotes from unclassified bins may support the presence of related uncharacterized taxa or new strain lineages of characterized species. Niche-specific and localized concentrations of eukaryotic microbes may exist across the gastrointestinal (GI) tract and between the mucoid and luminal compartments, highlighting the need for more targeted efforts in defining the eukaryome based on environmental context (18). We expect that continued use of unbiased methods with sufficient power to detect less abundant eukaryotes, as demonstrated here and recently by others (48, 68), will fill in gaps in the ecological framework in host-associated niches and have the potential to reveal the presence of other protists, uncharacterized fungi, and macro-eukaryotes in healthy humans.

Each microbiome sample harbored many diverse eukaryotic species. Reads assigned to taxa by read classifiers or by direct alignment to reference genomes support the existence of a large number of diverse eukaryotes in the human gut, including fungi and protists. All 10 of the most abundant fungal species detected in the HMP cohort were present in these two samples. Importantly, contigs greater than 1 kb could be independently constructed from reads for many of these species detected by the Kraken2 classifier. Among protists, the single-celled parasite *Blastocystis* was present in both individuals, suggesting it may be an extremely ubiquitous gut resident (68, 74). Singleton contigs often matched diverse protists that are not known to be present in the healthy human gastrointestinal tract (e.g., *Theileria equi*.). It is possible that reads from unsequenced microbes are being assigned to other species and producing low count matches for many taxa that have identical sequences over short genomic regions to these presumably related microbial eukaryotes that have not been sequenced.

Characterization of gene content from sorted microbiome samples can provide insight into the molecular roles of fungi and protists in the human host. Reduced taxonomical diversity of microbial eukaryotes in humans has been argued to be a consequence of secondary roles in metabolism that require fewer specialized pathways (75). However, we note that this may reflect a limited understanding of the eukaryome in these contexts. MAG assembly from eukaryote and unclassified read groups yielded contigs that can be mined for gene function, which have proven particularly insightful in bacterial studies (76, 77). It is important to note that binning individual chromosomes into a single genome remains a major limitation in eukaryote MAG assembly, although pathway complementarity and other methods may aid genome assembly (78, 79).

Increasing access to study the eukaryome will facilitate the pursuit of new research questions and the ability to revisit prior associations between microbial eukaryotes and diseases that lack experimental support. For example, inflammatory bowel disease has been linked to either an increased prevalence of fungi or specific fungal species (80, 81), an increase in fungal diversity (82), or a decrease in fungal diversity (32). The use of amplicon-based techniques that bias the identification of certain taxa may underlie some of these discrepancies and could be resolved through metagenomic approaches to increase the detection of diverse eukaryotic microbes and establish absolute eukaryome quantification through the use of known microbial spike-ins. Ultimately, improved approaches for microbial eukaryotes in the microbiome will facilitate their inclusion in routine microbiome research and ecological understandings of a healthy microbiome.

### Limitations to the current study

This study addresses many of the deficits in current approaches to the study of the eukaryome but requires considering its limitations. First, DNA from some eukaryotic cell morphologies is less efficiently recovered than others. Fungal spores are both more challenging to lyse and tend to fall outside of the FACS gates defined here because of their small size. Second, differences in cell sorting and DNA recovery can lead to biases in taxonomical representation in the stool. This is a challenge in bacterial metagenomics as well (83) and can be at least partially addressed using consistent methods as we describe here to improve precision and facilitate across-sample comparisons. Finally, the construction of MAGs is limited by large eukaryotic genome sizes, the presence of multiple chromosomes in eukaryotic cells, and community complexity limits the ability to construct longer and more informative contigs. Improvements to this approach and deep sequencing may be able to overcome these current challenges and improve our definitions and understanding of the eukaryome.

## MATERIALS AND METHODS

### Fecal sample collection

Fecal samples were collected using the OMNIgene Gut (DNAgenotek, Ottawa, Ontario) collection kit. Samples were stored within two weeks of collection and stored at −80°C. Samples were shipped on dry ice from the Native BioData Consortium on the Cheyenne River reservation to The Ohio State University.

### Microbial culturing

*E. coli*, *S. aureus*, and *S. typhimurium* were grown in Luria-Bertani broth (LB) (10 g tryptone, 5 g yeast extract, and 10 g sodium chloride in 1 L of water) overnight at 37°C. *L. reuteri* was grown in MRS broth (BD Difco, Franklin Lakes, NJ) for 48 hours at 37°C. *C. difficile* was grown in Reinforced Clostridial Medium (RCM) (3 g yeast extract, 10 g lab-lemco powder, 10 g peptone, 5 g glucose, 1 g soluble starch, 5 g sodium chloride, 3 g sodium acetate, and 0.5 g cysteine hydrochloride in 1 L of water) overnight at 37°C with an atmospheric gas mixture of 5% H_2_, 15% CO_2_, and 85% N_2_. *A. fumigatus* was grown on Yeast Peptone Dextrose agar (20 g peptone, 10 g yeast extract, 50 mL of 40% glucose, 2.5 mL of 10 mg/mL uridine, and 20 g of agar in 1 L of water) for 4 days at 37°C. To isolate *A. fumigatus spores* from a fungal lawn, the plate was washed with a tween solution (10% tween 20 in 100 mL 1× PBS). Briefly, 10 mL of the tween 20 solution was added to the fungal lawn and gently scrapped with an L-spreader. After scraping the plate, the liquid was removed, centrifuged, and washed with 1× PBS. *C. albicans*, *C. neoformans*, *P. pastoris,* and *S. cerevisiae* were grown in Yeast Peptone Dextrose (YPD) medium (20 g peptone, 10 g yeast extract, 50 mL of 40% glucose, and 2.5 mL of 10 mg/mL uridine in 1L of water) overnight at 30°C. *L. major* was grown in 10 mL flasks in Modified M199 Medium (M199 supplemented with 10% heat-inactivated fetal bovine serum, 1% 10,000 µg/mL PennStrep, and 1% 1M HEPES) for 2–3 days at 27°C to reach confluency and shift the medium coloration from pink to orange. *T. brucei* was grown in 10 mL flasks with SDM-79 Medium (7 g MEM Powder, 2 g Grace’s Insect medium, 8 mL of 50× MEM essential amino acids, 6 mL of 100× MEM non-essential amino acids, 1 g glucose, 8 g HEPES, 5 g MOPS, 2 g sodium bicarbonate, 0.1 g sodium pyruvate, 200 mg DL-alanine, 100 mg L-arginine, 300 mg L-glutamine, 70 mg DL-methionine, 80 mg L-phenylalanine, 600 mg L-proline, 60 mg DL-serine, 160 mg taurine, 350 mg DL-threonine, 100 mg L-tyrosine, 10 mg guanosine, 4 mg folic acid, 50 mg D(+)glucosamine hydrochloride, 2 mg p-aminobenzoic acid, and 0.2 mg biotin in 1L of water) at 37°C for 2–3 days to reach confluency. *C. elegans* was grown on solid Nematode Growth medium (3 g NaCl, 2.5 g peptone, 1 mL of 1 M CaCl_2_, 1 mL of 5 mg/mL cholesterol in ethanol, 1 mL of 1 M MgSO_4_, 25 mL of 1 M KPO_4_, and 17 g agar in 975 mL of water) with a lawn of OP50 *E. coli* as a food source at 27°C for 3 days.

### *C. elegans* egg isolation

To isolate eggs, *C. elegans* was grown to the adult gravid stage as previously described and removed from the plate by rinsing the plate with 10 mL of M9 buffer (70 g Na_2_HPO_4_.7H_2_O, 30 g KH_2_PO_4_, 5 g NaCl, and 10 g NH_4_Cl in 1 L of water). The worms were pelleted by centrifuging for 1 minute at 1,500 rpm at room temperature. The supernatant was gently removed, and 8.7 mL of hypochlorite solution (400 µL 5M NaOH, 1 mL bleach, and 6 mL of water) was added, gently vortexed, and incubated for 5 minutes. After the incubation period, 7 mL of M9 buffer was added and then centrifuged for 1 minute at 1,500 rpm. After centrifugation, the supernatant was removed, 14 mL of M9 buffer was added, and the suspension was centrifuged for 1 minute at 1,500 rpm. The washing and centrifugation steps were repeated three times. After the washes, 500 µL of M9 buffer was used to resuspend the isolated eggs and remaining intact adult worms.

### Development of an optimized eukaryotic lysis protocol

All optimization steps were performed with overnight YPD cultures of *C. neoformans*. Cells were washed with saline twice, enumerated *via* hemocytometer, and spiked into fecal samples with 10^7^ cells. To optimize cell separation from fecal matter, 1 mL of 0.9% saline solution was added to the fecal matter and vortexed for 10 seconds. Three parameters were subsequently tested: centrifugation speed, gravity separation, and sonication. For centrifugation speed, samples were centrifuged between 100 and 400 × *g* and the supernatants were collected; this was repeated four times. For gravity separation, spiked fecal samples sat undisturbed at room temperature for 10 minutes for fecal particulate settlement or centrifuged at 300 × *g* for 3 minutes; this was repeated four times, and supernatants were collected. Finally, to test sonication, fecal samples were placed in a water bath sonicator for 1 minute, and both undisturbed and sonicated samples were centrifuged for 3 minutes at 300 × *g*x to collect the supernatant. Isolation of GFP^+^ *C. neoformans* from feces in the collected supernatants was determined using a BioTek Synergy H1 Microplate Reader (Agilent Technologies, Winooski, VT) set at excitation and emission wavelengths of 485 and 528 nm, respectively. Analysis was performed using BioTek Gen5 software.

Once washing conditions were determined, four other species were tested: *C. albicans*, *P. pastoris*, *L. major*, and *C. elegans*. Yeast species were grown in overnight YPD cultures. *L. major* was grown in a 3-day in modified M199 media. Yeast and protozoan cultures were washed with saline twice, enumerated with a hemocytometer, and 10^7^ cells spiked into 200 mg of human feces. Each fecal sample was washed with 1 mL of 0.9% saline, vortexed for 10 seconds, centrifuged for 3 minutes at 300 × *g*, and supernatant gently collected. The above steps were repeated three more times. Fluorescence measurements were performed using a BioTek Synergy H1 Microplate Reader. Isolation of DAPI-stained *C. albicans* from feces in collected supernatants was determined using the microplate reader set at excitation and emission wavelengths of 350 nm and 465 nm, respectively. Isolation of GFP^+^ *P. pastoris* from feces in collected supernatants was determined using the microplate reader set at excitation and emission wavelengths at 485 nm and 528 nm, respectively. Isolation of mCherry^+^ *L. major* from feces in collected supernatants was determined using the microplate reader set at excitation and emission wavelengths of 570 nm and 610 nm, respectively. Analysis was performed using BioTek Gen5 software.

*C. elegans* was grown on Nematode Growth Media with an *E. coli* lawn, and eggs were isolated using the hypochlorite procedure. From growth assays, 22,000 eggs were collected and resuspended in 500 µL. Vortexing the eggs, the 500 µL sample was split into 5 100 μL aliquots, and each fecal sample received an individual 100 μL inoculation of *C. elegans*. Each fecal sample was washed with 1 mL of 0.9% saline, vortexed for 10 seconds, centrifuged for 3 minutes at 300 × *g*, and supernatant gently collected. The above steps were repeated three more times. Each collected supernatant was extracted using the methodology developed for this manuscript. Purified DNA was amplified for the tmeel-10 gene using the following reaction mix: 4 mL of Phusion HF Buffer, 5× concentrated; 0.5 µL 10 mM dNTPs, 1 mL of each forward (5′- CTCAACTACGGTTGCCAATGC-3′) and reverse primers (5′-AGTTAGCCAGTACAGCGGAC-3′) (10 mM each), 8.4 mL of water, 0.2 μL of Phusion polymerase, and 5 mL of DNA template. Amplification was performed in an Applied Biosystems PCR System using the following cycling conditions: 1 minute at 98°C followed by 35 cycles of 15 s at 98°C, 30 s at 57°C, and 45 s at 72°C. PCR amplicons were run on a 1% gel and analyzed using Image Lab 5.2.1 to determine DNA concentration for *C. elegans* gDNA in each gel well.

Optimization of cell disruption occurred in four phases using 10^7^ purified *C. neoformans* cells: size of beads, volume of beads, duration of bead beating, and buffer. For bead size determination, cells were added to 2.0 mL screw-cap tubes with one of five bead types at 300 mg: no bead control, 100 nm glass beads, 500 nm glass beads, 1,000 nm glass beads, and glass shards. Glass shards were made by pulverizing 1,000 nm beads into jagged, smaller pieces with a mortar and pestle. Cells were bead beat for five cycles (1 minute on, 2 minutes off) with a modified Mini-Beadbeater-8 (Biospec, Bartlesville, OK). For bead volume optimum, aliquots of 0 to 800 mg of 500 nm beads were added with *C. neoformans* to 2.0 mL screw-cap tubes and bead beat for five cycles (1 minute on, 2 minutes off). To assess the duration of bead beating, *C. neoformans* cells were transferred to 2.0 mL screw-cap tubes filled with 600 mg of 500 nm glass beads and beaten for 0 to 8 cycles (1 minute on, 2 minutes off) with tubes imaged after each cycle. 10 µL from each lysis sample was removed and added to a fresh microcentrifuge with 10 µL of trypan blue 0.4% solution (Thermo Fisher Scientific, Waltham, MA). The sample was mixed by gently pipetting up and down, and 10 mL was visualized by microscopy using a Leica DM 750 with Leica MCD170 HD Camera at 40× magnification. Images were processed using Leica Application Suite 4.12.0 software. The percentage of lysed cells was determined by counting the number of intact versus lysed cells indicated by the entrapment of trypan blue dye (lysed) per each field of view. To test buffers, *C. neoformans* was resuspended in five different lysis buffers in 2.0 mL screw-cap tubes: TENT [10 mM tris-HCl, 1 mM EDTA, 0.1 M sodium chloride, 5% (vol/vol) triton X100, pH 8), TENTS (TENT +3% SDS), cetyl trimethyl ammonium bromide (CTAB), Qiagen Powersoil buffer, and Epicenter Masterpure Yeast DNA Purification buffer. Cells were bead beat for 5 cycles with 500 mg of 500 nm glass beads. DNA concentration was measured with Qubit 4.

### Comparison of fungal DNA recovery

Fungal DNA recovery was performed using overnight cultures of *C. neoformans* that were diluted to 1.0 × 10^7^ cells/mL, washed with 1 mL of 1× PBS, and resuspended in 1 mL of sterile synthetic stool. *S*amples were extracted for DNA using the following kits according to the manufacturer’s instructions and included bead-beating steps: Epicenter Masterpure Yeast DNA Purification Kit (cat #MPY80200), OMNI Fecal DNA Purification Kit (cat #26–014B), ZymoBIOMICS DNA Microprep Kit (cat #D3400), and Qiagen Powersoil Pro Kit (cat #47014). *S*amples were also extracted using bead beating in TENT buffer [10 mM tris-HCl, 1 mM EDTA, 0.1 M sodium chloride, and 5% (vol/vol) triton X100, pH 8] for 5 minutes with 500 nm beads as described above.

### DNA extraction and quantification

An addition of 300 µL of Phenol:Chloroform:Isoamyl Alcohol (25:24:1) (Sigma-Aldridge) and 200 µL of 1× TE was added to the 700 µL of buffered lysed cells, and the tubes were rocked back and forth to fully homogenize the mixture. The cell-phenol mixture was centrifuged at 4°C at full speed for 10 minutes, the aqueous layer was removed, and this layer was mixed with an equal volume of Chloroform:Isoamyl Alcohol (24:1). The chloroform-aqueous tube was rocked to homogenize and then centrifuged at full speed for 10 minutes at 4°C. After the chloroform-aqueous centrifugation, the aqueous material (top layer) was removed and mixed with 3× volume of chilled 95% ethanol and 1/10th volume of 3M sodium acetate. The aqueous-ethanol mixture was allowed to incubate at −20°C for at least 1 hour. After the −20°C incubation, the aqueous material is centrifuged for 20 minutes at 12,000 rpm and resuspended in 50 µL of water. Subsequent DNA purification was carried out with in-house produced magnetic beads at 1.5× volume (150 mL) to remove potential contaminants. Briefly, magnetic beads (GE Sera-Mag Carboxylate Modified Speed Beads) were incubated with the DNA for 10 minutes, allowing for maximal DNA binding. The bead-DNA mixture was added to a magnetic rack to form a pellet, and the beads were washed with 80% ethanol. Ethanol was removed and the bead-DNA pellet was allowed to dry for 5 minutes. 50 mL of water was added to the DNA-bound beads, allowing for the DNA to be removed from the beads for 5 minutes. The mixture was added to the magnetic rack for 3 minutes, and then the DNA was removed. DNA concentration and purity were measured using a Qubit 4.0 Fluorometer. DNA purity was determined *via* the 260/280 and 260/230 ratios measured on the Nanodrop one.

### Extraction efficiency

Extraction efficiency was performed using overnight cultures of *C. albicans* and *S. cerevisiae*, washed with saline twice, enumerated with a hemocytometer, diluted to 2 × 10^7^ cells, and extracted using the above extraction methodology. A third yeast, *P. pastoris* was grown overnight, washed with saline twice, enumerated with a hemocytometer, and diluted to 1 × 10^8^ cells. *Aspergillus fumigatus* extraction efficiency was performed on both spores and hyphae. Briefly, *A. fumigatus* spores were harvested as previously mentioned, washed with saline twice, enumerated with a hemocytometer, and extracted 1 × 10^7^ spores. *A. fumigatus* hyphae were extracted by harvesting spores, washed with saline twice, enumerated with a hemocytometer, diluted to 1 × 10^7^ spores, and inoculated into 2 mL of liquid YPD. Spores were incubated at 37°C for 24 hours. Spores and hyphae were extracted using the methodology mentioned above. *L. major* extraction efficiency was performed using a 3-day culture, washed with saline twice, enumerated with a hemocytometer, diluted to 2 × 10^7^ cells, and extracted using the above extraction methodology. The DNA was quantified using a Qubit 4 Fluorometer. Extraction efficiency was calculated using the following equations:


Total DNA=Ploidy × Mbp

$$Total\;DNA\;in\;g=(Total\;DNA\;\times \;650)/(6.022\times 10^{23})$$

Total DNA in cell pellet=(Total DNA in g × Cell Numbers)

$$Convert\;g\;to\;ng\;=Total\;DNA\;in\;cell\;pellet\;\times \;10^{–9}$$

Extraction Efficiency=(ng/μL × DNA vol)/(Total DNA in cell pellet ng)

### Methylated restriction endonuclease activity

1 μg of *E. coli* and *C. neoformans* DNA were digested individually and together in the presence of 1 U DpnI (NEB) and Cutsmart buffer (NEB) for 3 hours at 37°C. DNA digestion was visualized on 2% TAE agarose gel. To assess which magnetic bead concentration was optimal for eukaryote recovery, three concentrations were tested: 0.7×, 0.5×, and 0.4×, as described above.

### Mock community engineering and extraction

A mock community was engineered by inoculating 10 different species into a synthetic stool (ClaremontBio, cat# 01.381.80). Briefly, cultures of 5 eukaryotes (*C. albicans, C. neoformans, S. cerevisiae, L. major, T. brucei*) and five bacteria (*C. difficile, E. coli, L. reuteri, S. typhimurium,* and *S. aureus*) were grown and counted *via* a hemocytometer or OD_600_, respectively. For mock communities with a 1:1 ratio of bacteria to eukaryotes, 10^7^ cells of each species were inoculated into 1 mL of sterile synthetic stool. For mock communities with a 99:1 ratio of bacteria to eukaryotes, 10^10^ bacterial cells, and 10^8^ eukaryotic cells were inoculated into 1 mL of sterile synthetic stool. The mock communities were then frozen at −20°C. Mock communities were extracted for DNA as specified above. To determine DpnI digestion performance in the mock community, 200 µg of DNA was digested with 1 U DpnI for 3 hours at 37C. DNA from the mock community was analyzed using qPCR conditions specified below.

### qPCR of microbial species

Purified DNA was quantified by amplifying the following reaction mix: 6 mL of PowerUp SYBR Green Master Mix, 2× concentrated (Applied Biosystems, Waltham, MA); 1 mL of each forward and reverse primers (10 mM each, see Table S1), 3 mL of water, and 1 mL of DNA template. Amplification was performed in an Applied Biosystems QuantStudio Real-Time PCR Systems model 3 using the following cycling conditions: 2 minutes at 50°C and 5 minutes at 98°C followed by 40 cycles of 15 s at 98°C and 30 s at 56-58°C. Quantification was determined against a standard dilution series generated for each organism in the mock community.

### Microbial eukaryote enrichment

To establish FACS gates, GFP^+^ *C. neoformans* and RFP^+^ *S. aureus* were grown overnight in liquid YPD and LB, respectively. Cells were washed with saline twice and resuspended in 1 mL of 0.9% saline solution. GFP^+^ *C. neoformans* and RFP^+^ *S. aureus* was sorted individually and in combination using a FACS Aria III (BD Biosciences, Franklin Lakes, NJ), and forward and side scatter regions of interest were established to delineate each species. Once the gates were determined using the above controls, GFP^+^ *C. neoformans* was spiked into a 200 mg fecal sample at 1 × 10^7^ cells in 100 µL. The fecal sample was diluted in 1 mL of 0.9% saline solution, vortexed for 10 seconds, and then centrifuged for 3 minutes at 300 × *g* speed. The supernatant was collected, and this process was repeated three more times. Cells from fecal washes were sorted into separate collection tubes using this gating strategy for the entire processed sample. Collected cells were pelleted at 3,900 rpm for 30 minutes, and DNA was extracted using the optimized protocol developed here. All FACS analysis was performed using FlowJo software v8.7.

To verify that other microbial eukaryotes are captured in the gating strategy developed above, 11 different microbes were individually cultured and then sorted: *C. difficile, S. typhimurium, A. fumigatus hyphae, A. fumigatus spores, C. albicans* hyphae*, C. albicans* yeast*, P. pastoris, S. cerevisiae, L. major, T. brucei,* and *C. elegans*. Each culture was sorted for 200,000 events on a FACS Aria III (BD Biosciences, Franklin Lakes, NJ). Briefly, each organism (except *C. elegans*) was cultured alone, diluted to 1 × 10^7^ cells, washed with 1× PBS, and resuspended in 1 mL of 1× PBS.

To isolate hyphae, an overnight liquid culture of *C. albicans* was washed with 1× PBS, diluted to 1 × 10^7^ cells, and suspended in 100 μl of YPD. The 100 μl suspension was added to 3 mL of liquid YPD and incubated for 3 hours at 37°C. Hyphae development was confirmed *via* a microscope visualization. *A. fumigatus spores* were isolated from a fungal lawn grown on a YPD plate for 5 days and washed with a tween solution (10% tween 20 in 100 mL 1× PBS) to remove the spores. Spores were washed with 1× PBS, diluted to 1 × 10^7^ spores, and resuspended in 100 μl of liquid YPD. The cultures were allowed to incubate for 1–2 days at 37°C. Once a fungal mass developed, the mass was removed and pulverized with 200 mg of glass beads for 1 minute to break up hyphae.

*C. elegans* was grown and harvested for eggs, as mentioned previously. Eggs and residual adult gravid worms were fixed with a 1 mL solution of 1× PBS and 4% paraformaldehyde solution for 1 hour on a rotator; post-incubation, eggs were placed immediately on ice and sorted for 5000 events on FACS Aria III. All FACS analysis was performed using FLowJo software v8.7

To determine the efficacy of sorting mixed culture samples, *S. typhimurium* was mixed with *C. albicans*, mCherry^+^ *L. major*, and GFP^+^ *P. pastoris*. Each culture was individually grown, washed with 1× PBS, counted *via* a hemocytometer, and resuspended in 1 mL. Microbial eukaryotes were diluted to 1 × 10^7^ cells/mL, and *Salmonella* was diluted to 1 × 10^9^ cells/mL. 200,000 events were sorted using FACS Aria III and analyzed using FlowJo software v8.7. *C. albicans* cells were pre-stained with DAPI dye that labels the cell wall.

### Fecal metagenomic sequencing

DNA was extracted from 200 mg of each fecal sample using either the Qiagen Powersoil Kit including the bead-beating steps (Q), the optimized bead-beating protocol (B), the bead-beating protocol and enrichment (BE), and the FACS sorting microbial eukaryotes combined with the developed bead-beating protocol (FACS). For each sample, 100,000 events were captured in Gate 1 and Gate 2 for the sorted pools prior to beginning cell lysis and DNA isolation. Sequencing libraries were constructed using the NEBNext Ultra II FS DNA Library Prep Kit according to the manufacturer’s instructions. The kit performs well when beginning with low biomass and does not require any special considerations during library construction. Libraries were quantified using a Qubit 4.0 Fluorimeter and checked for fragment distributions with an Agilent 2100 Bioanalyzer using a High Sensitivity DNA Kit. Libraries were pooled in equimolar ratios and sequenced on an Illumina Hiseq 4000 as 150 bp paired-end reads (Table S2). Sequenced libraries were assessed with FastQC and trimmed with Trimmomatic v0.36 (84, 85). Human reads were filtered out sequences using BBDuk and alignment to the human reference genome hg38 (86). The remaining reads were merged with PANDAseq v2.11 and taxonomically assigned using Kraken2 v2.1.2 (87, 88). For comparison to the Human Microbiome Project, merged reads from each library were aligned using Bowtie2 (89). Briefly, FACS reads from samples 21 and 25 with a minimum length of 50 bp were aligned with the 10 fungal species (*Malassezia restricta, S. cerevisiae, Malassezia globosa, Cyberlindnera jadinii, Saccharomyces pastorianus, C. albicans, Debaryomyces hansenii, Malassezia sympodialis, Alternaria alternata*, and *Candida parapsilosis*) identified to be the most prevalent fungi in the gut by Nash et al. (48). Bowtie alignments were restricted to perfect matches of the full-length sequence. To calculate fold change performance between HMP- and FACS-enriched samples, FACS sample Bowtie2 alignment outputs were combined, total read numbers combined, a relative abundance of fungal read calculated for HMP and FACS-processed samples, and then divided by HMP fungal read relative abundance.

### Gene predictions for microbial eukaryotes

MAGs (Metagenome Assembled Genomes) were built with MEGAHIT v1.2.9 (90). MAG contigs below 1,000 bp were subsequently removed using a custom script. Using the curated MAG fasta file, genes were annotated using Augustus v3.5.0 and Diamond v2.0.15 (91, 92). Model organisms used for gene predictions in Augustus included *Toxoplasma gondii, Schistosoma mansonii, Botrytis cinerea, C. albicans, Candida tropicalis, Candida guilliermondi, C. neoformans, D. hansenii, Pichia stipitis, S. cerevisiae,* and *Ustilago maydis*. After Augustus predictions, Diamond identified and annotated the Augustus (.gff file) outputs using the NCBI non-redundant protein database. Final gene predictions removed all bacterial, mammalian, avian, arachnid, insect, lizard, *C. neoformans*, and *Trichuris* gene predictions, all hits below 90%, and duplicates.

### Figures and statistics

Each experiment was performed with at least three biological replicates, with three technical replicates each. Statistical tests were performed in GraphPad Prism 8 (one-way or two-way ANOVA, *t*-test, and/or Tukey’s post hoc test) or StataIC (Pearson’s correlation and linear regression model tests). Biorender was used to design figures within this manuscript.

## Data Availability

All high-level data generated or analyzed during this study are included in this published article (and its supplementary information files). The data sets generated and/or analyzed during the current study are available from the corresponding author or the Native BioData Consortium on request and with the permission of the Native BioData Consortium as a mediator of tribal sovereignty with the Cheyenne River Sioux Tribe.

## SUPPLEMENTAL MATERIAL

Supplemental material mbio.02063-24-s0001.pdf Supplemental figures and tables and a detailed protocol.

ASM does not own the copyrights to Supplemental Material that may be linked to, or accessed through, an article. The authors have granted ASM a non-exclusive, world-wide license to publish the Supplemental Material files. Please contact the corresponding author directly for reuse.
