# Supplementary material for: Metagenomic discovery of microbial eukaryotes in stool microbiomes
Source: mBio. 2024 Aug 29;15(10):e02063-24. doi: 10.1128/mbio.02063-24 (PMC11481512; doi:10.1128/mbio.02063-24)
Supplement: Supplemental material — Supplemental figures and tables and a detailed protocol. [file mbio.02063-24-s0001.pdf]

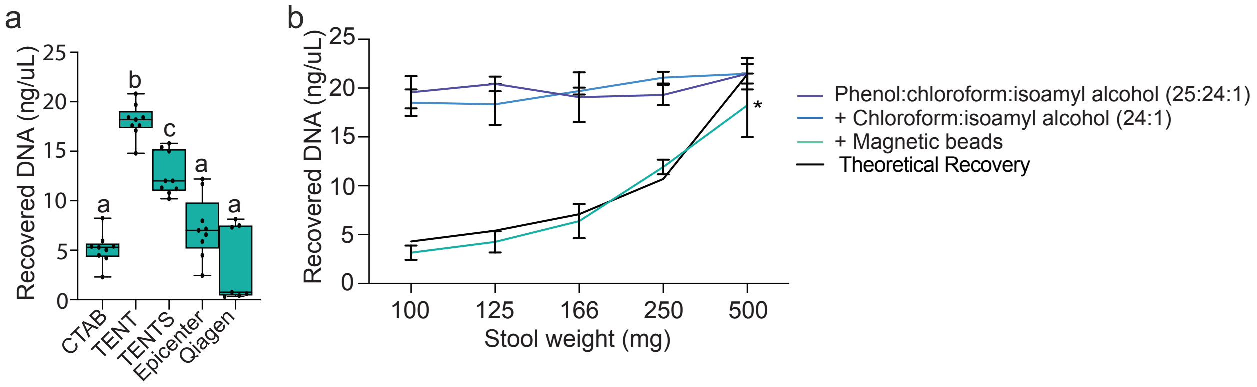

Supplemental Figure 1. Optimization of DNA recovery from synthetic stool seeded with fungi. a. C. neoformans cells seeded in synthetic stool (1x108 cells in 500 mg) were suspended in 500 L of various buffers commonly used in DNA extraction from microbiome samples. DNA recovery was plotted for each buffer. Boxplots mark the interquartile ranges and the median value with whiskers extending to the outermost data points. Letters indicate statistically different groups based on a one-way ANOVA and Tukey's posthoc test at $p < 0.05$. N = 9. **b.** DNA was extracted from different volumes of synthetic stool in TENT buffer seeded with 1x108 C. neoformans cells. The percentage of maximal DNA recovery is plotted when extracted and purified with either phenol:chloroform (purple), phenol:chloroform and an additional chloroform purification (blue), or phenol:chloroform, an additional chloroform purification, and magnetic bead purification (green). The black line dashed line indicates the maximal theoretical recovery. Data is plotted as averages with standard deviation. N = 3.

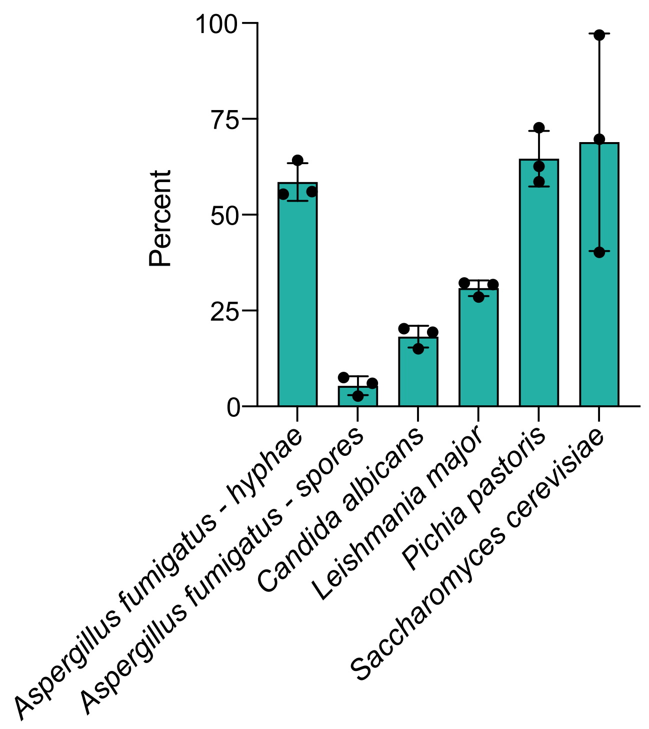

Supplemental Figure 2. DNA recovery of microbial eukaryotes by bead beating. Cells from each indicated species were lysed and the DNA recovered using the developed approach. The percent yield based on the calculated amount of DNA present in the initial microbial populations is plotted. The average is plotted with the standard deviation for each group. N=3.

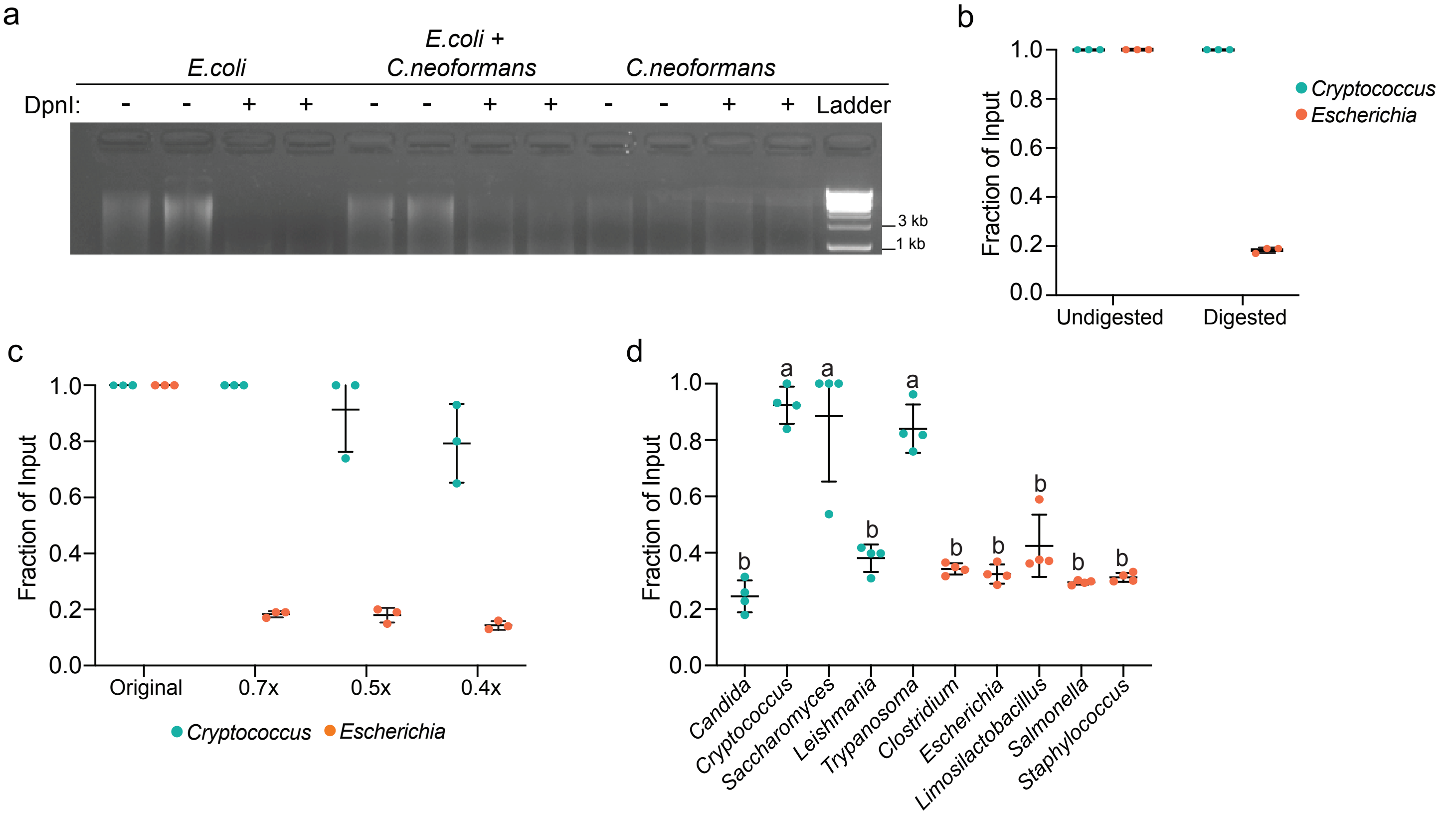

Supplemental Figure 3. Methyl-sensitive restriction enzymes digest bacterial but not eukaryotic DNA. a. Two 1 µg aliquots of E. coli (left), C. neoformans (right), or a 50:50 mix of E. coli:C. neoformans (center) DNA was incubated with 1 U of DpnI restriction enzyme or mock treated for 1 hour and run by gel electrophoresis. DNA sizes are marked by a 10 kb ladder. **b.** E. coli and C. neoformans DNA mixed in equal amounts was digested with 1 U DpnI or mock treated for 3 hours and recovered using 0.7x magnetic beads. The fraction of input DNA recovered is plotted. N = 3. **c.** DNA was digested using 1 U DpnI and purified with varying concentrations of magnetic beads. The fraction of recovered to input DNA is plotted as the average and standard deviation. N = 3. **d.** DNA from the 99:1 bacterial-dominant community was extracted and digested with 1 U DpnI for 1 hour. DNA abundance for each species was quantified by qPCR using species-specific primers and the ratio plotted. Data is presented as mean with standard deviation. N=4. The center line marks the median with whiskers extending to the outermost data points. Letters indicate statistically different fractions recovered based on a one-way ANOVA and Tukey's posthoc test at $p < 0.05$.

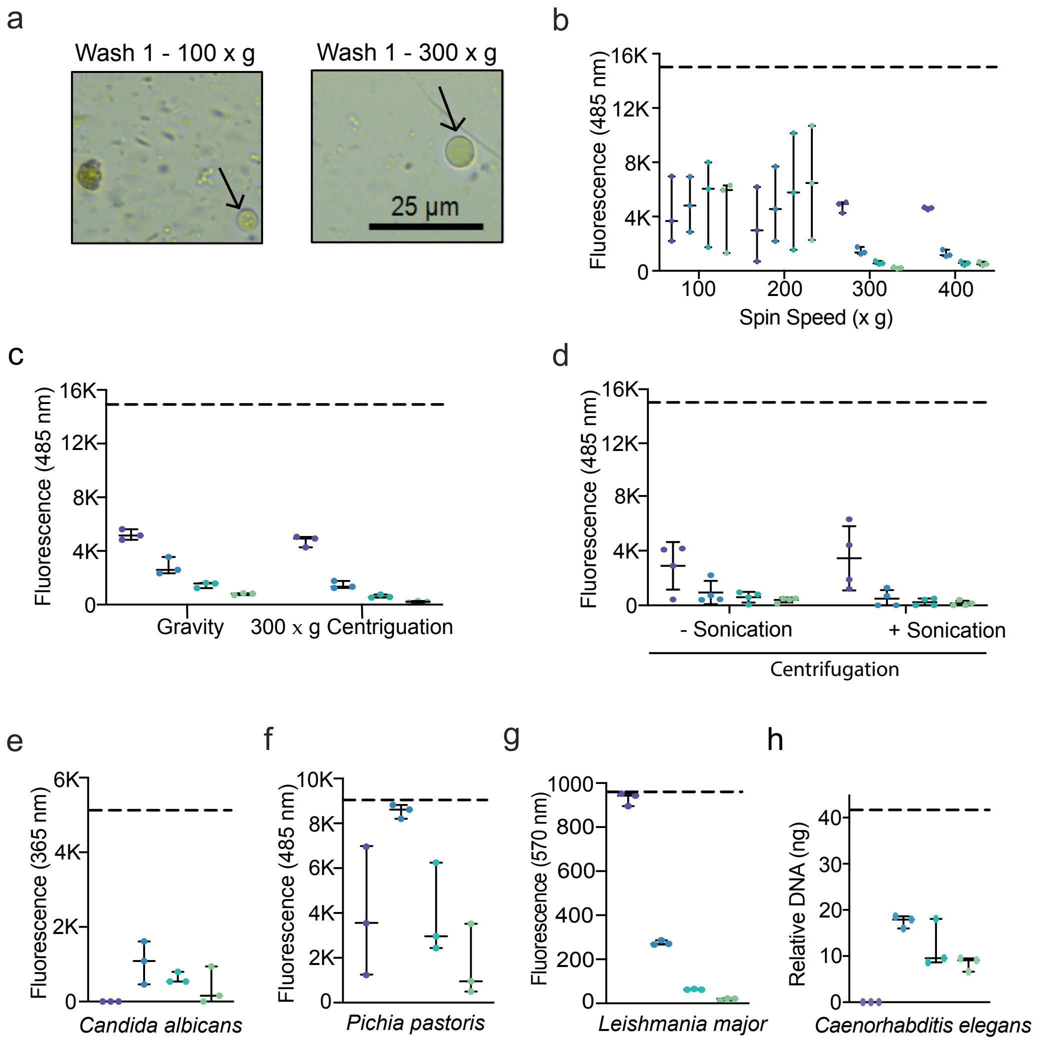

Supplemental Figure 4. Optimized recovery of microbial eukaryotes from fecal samples. a. Determination of the optimal conditions for microbial cell extraction from feces was performed by inoculating 200 mg of human adult fecal matter with 1 x 10^7 cells/mL of C.neoformans. Fecal material was centrifuged at either 100 x g or 300 x g for 3 min. The supernatant was removed and visualized at 40x magnification. Arrows indicate morphologically identifiable fungal cells. Scale bar = 25 µm. **b.** Fecal samples were vortexed for 10 seconds and centrifuged at 100, 200, 300, and 400 x g for 3 minutes. The process was repeated four times and the supernatant was collected from each sequential wash as indicated. The fluorescence of GFP+ C. neoformans in each wash is plotted. N=3. **c.** Fecal samples were either prepared using 300 x g centrifugation or allowed to settle by gravity for 10 minutes. The fluorescence of GFP+ C. neoformans in each wash is plotted. N=3. **d.** Fecal samples were either sonicated for one minute or left untreated prior to centrifugation for 3 minutes at 300 x g. Supernatant were collected after each centrifugation and the fluorescence determined to detect GFP+ C. neoformans. N=4. Retention of C. albicans (**e**), P. pastoris (**f**), L. major (**g**), and C. elegans eggs (**h**) in the supernatant was assessed for stool seeded with each organism during successive 300 x g centrifugations for 3 minutes each. Quantification was performed by staining the cell wall (C. albicans), GFP fluorescence (P. pastoris), mCherry fluorescence (L. major), or DNA quantification. N=3. Dashed lines indicate the maximum theoretical yield. The center line marks the mean with whiskers extending to the outermost data points.

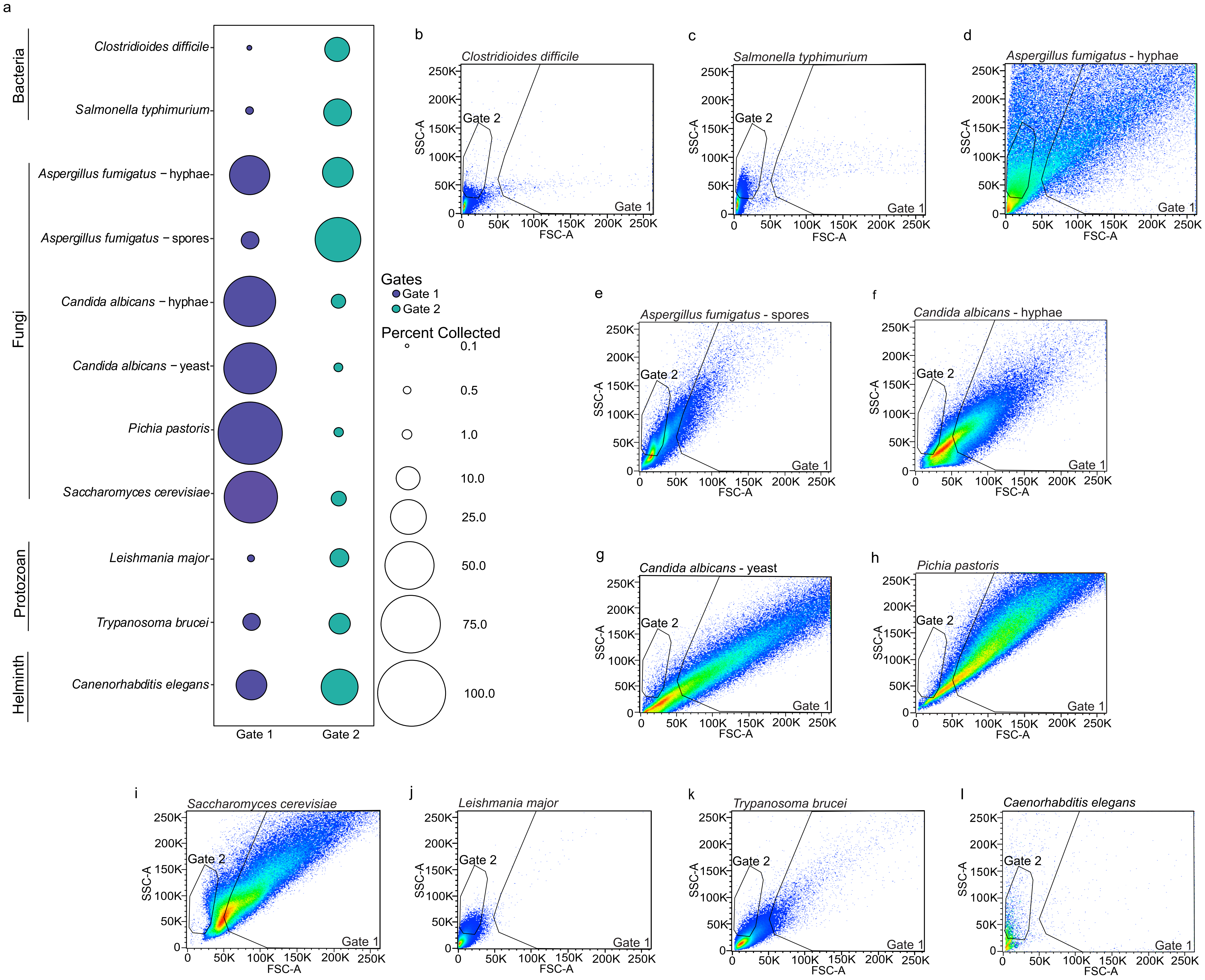

Supplemental Figure 5. Quantification of microbes in constructed FACS gates. a. A cell suspension of each microbe was produced and FACS sorted using the previously defined regions of forward and side scatter, designated as Gate 1 and Gate 2. The percentage of each pure microbial population located in Gate 1 and Gate 2 is indicated by the size of the circle. The FACS plots for C. difficile (**b**), S. typhimurium (**c**), A. fumigatus hyphae (**d**), A. fumigatus spores (**e**), C. albicans hyphae (**f**), C. albicans yeast (**g**), P. pastoris (**h**), S. cerevisiae (**i**), L. major (**j**), T. brucei (**k**), and C. elegans eggs and remaining adult worms (**i**) is given.

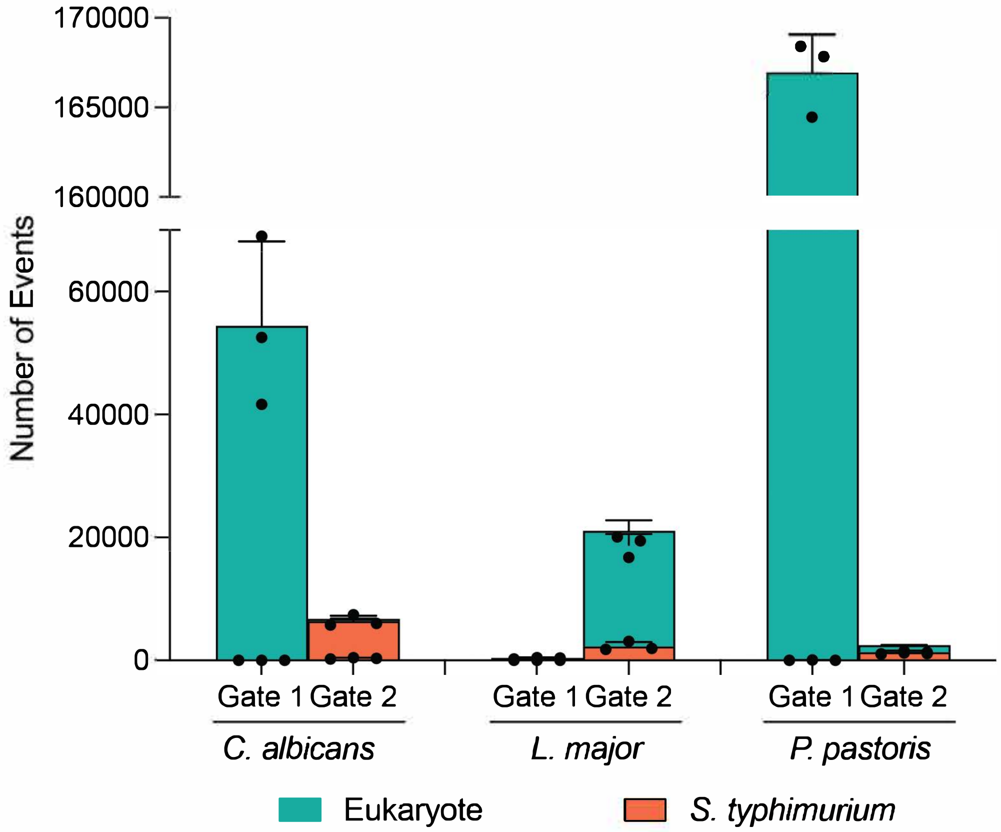

Supplemental Figure 6. Eukaryotic microbes are enriched in FACS gates. 1 x 10^7 fluorescently labeled eukaryotic cells and 1 x 10^9 S. typhimuirum cells were mixed and 200,000 events assessed by FACS. The number of events in Gate 1 and Gate 2 were quantified for each species based on fluorescence. The average is plotted with the standard deviation for each group. N=3.

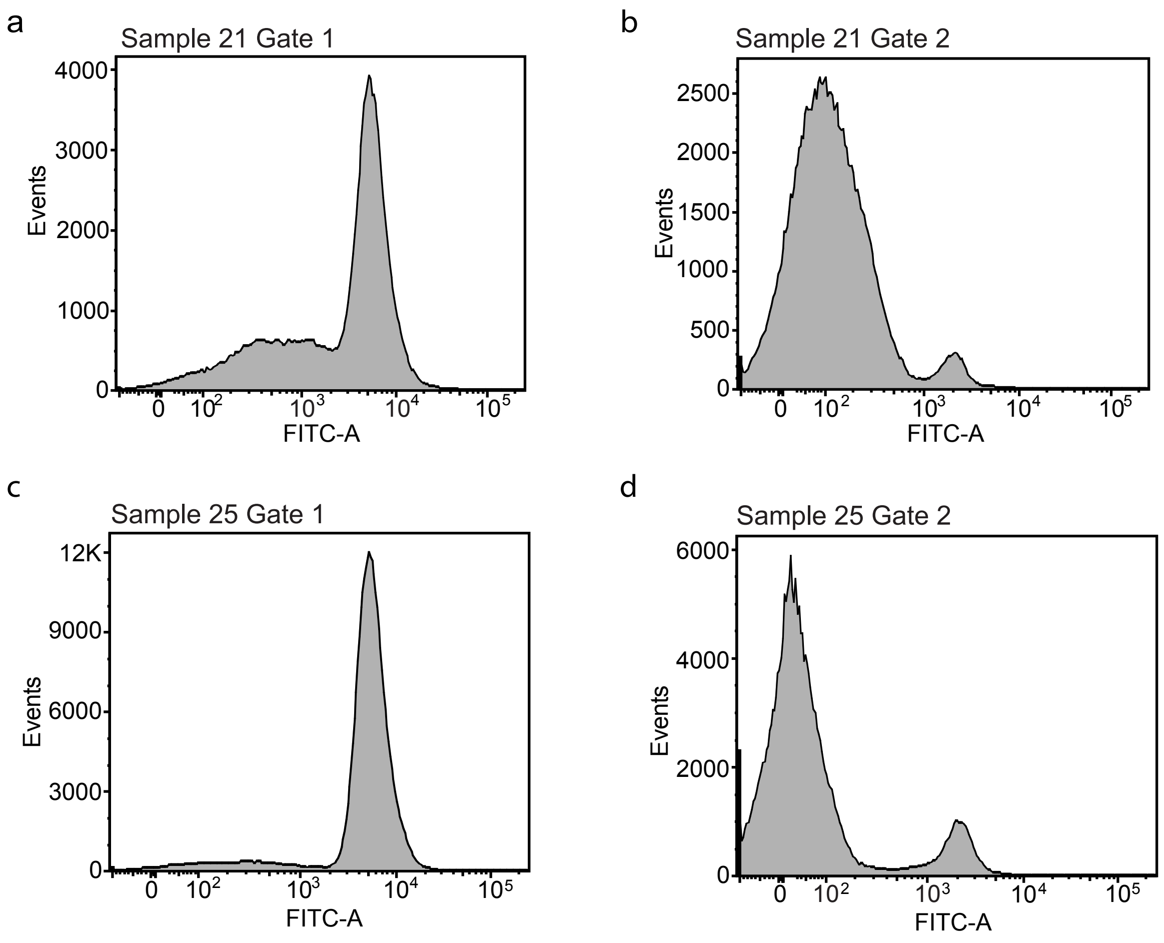

Supplemental Figure 7. Distribution of GFP+ events in fecal samples using constructed FACS gates. Stool was spiked with GFP+ C. neoformans and cells isolated by FACS using the previously established Gate 1 and Gate 2. The distribution of GFP fluorescence in 100,000 events isolated from sample 21 for Gate 1 (**a**) and Gate 2 (**b**) and sample 25 for Gate 1 (**c**) and Gate 2 (**d**).

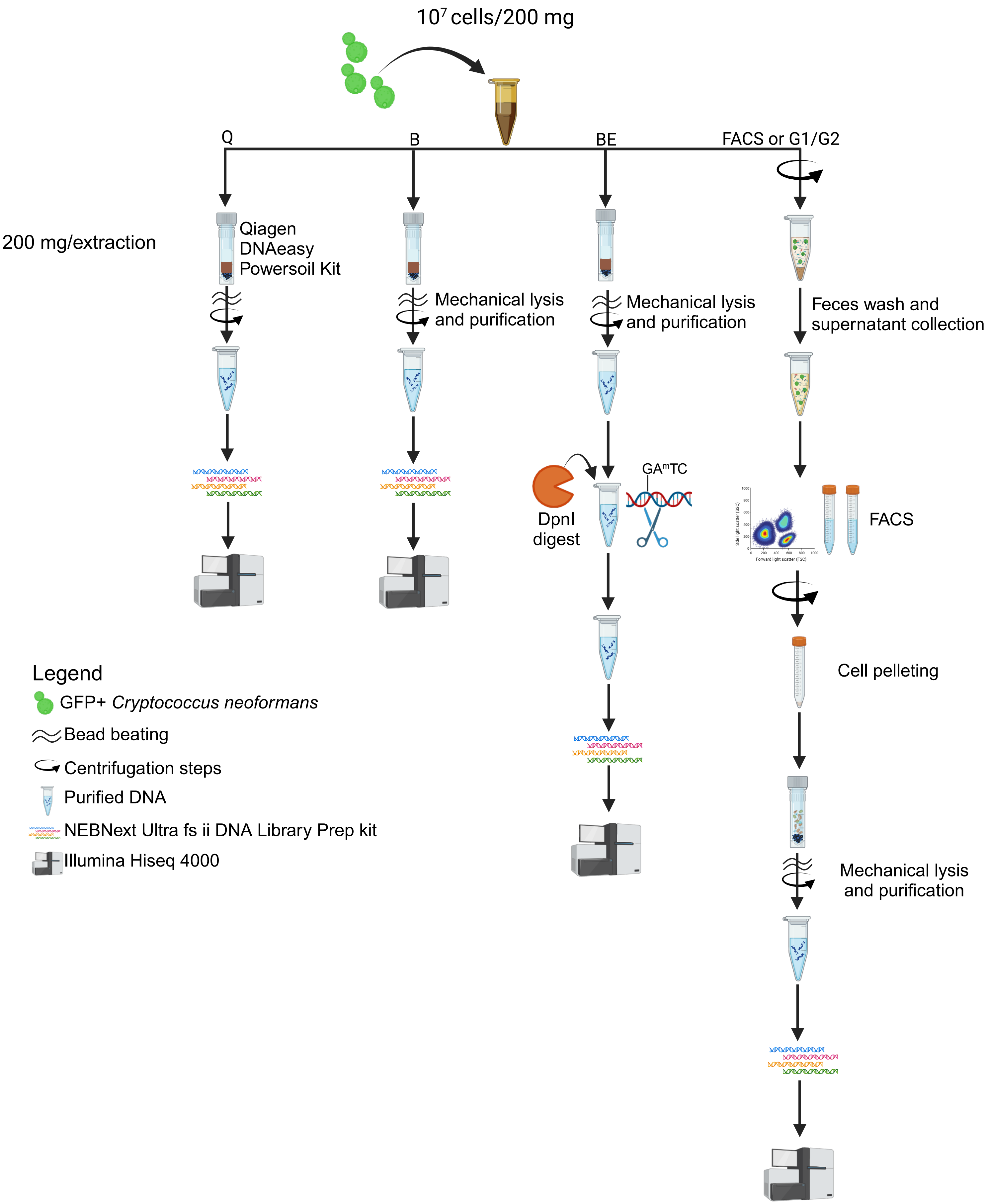

Supplemental Figure 8. Workflow for the comparison of metagenomic sequencing. A cartoon depiction outlining the steps taken for each approach taken for metagenomic sequencing of samples 21 and 25. Q denotes Qiagen Powersoil Extraction kit. B denotes bead beating. BE denotes bead beating and DpnI enrichment. FACS denotes use of the constructed gates and cell sorting.

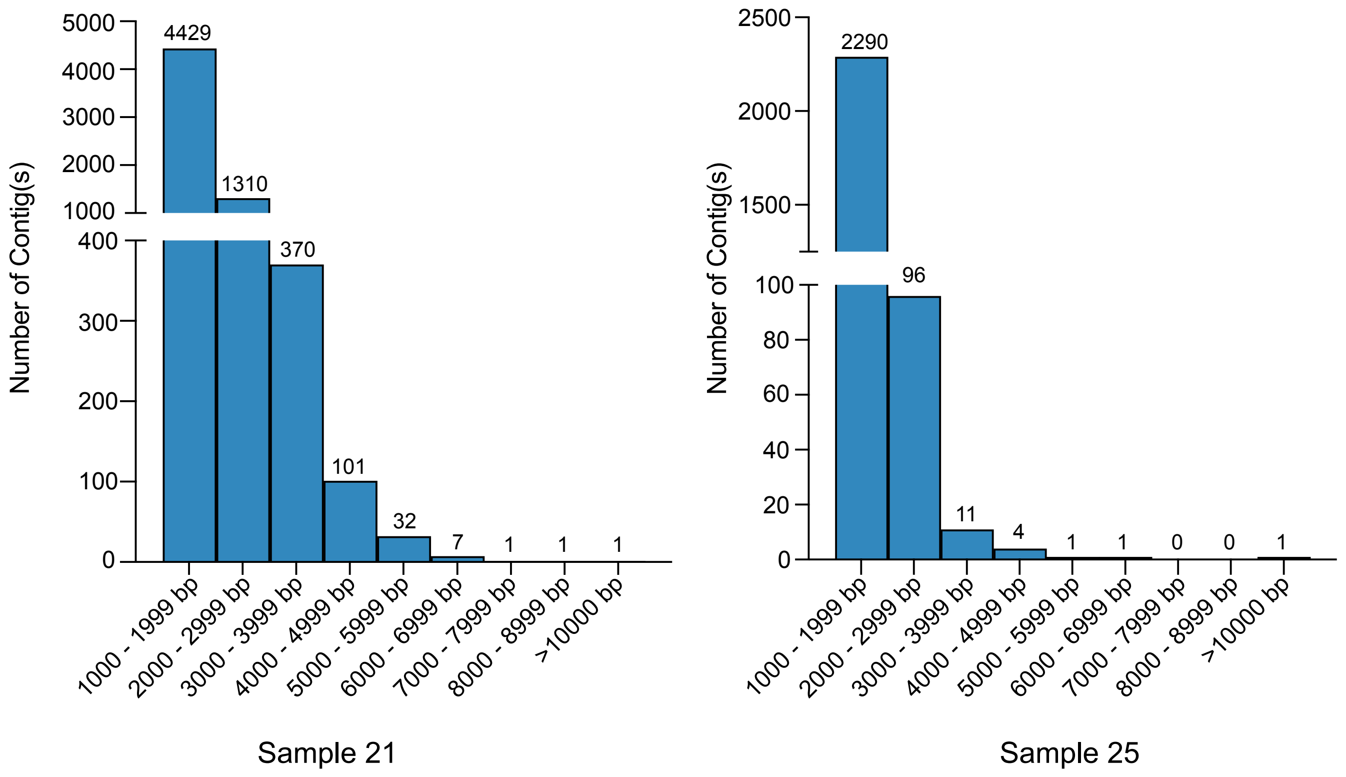

Supplemental Figure 9. C. neoformans contig assembly from eukaryote enriched sequenced pools. The number of contigs constructed from C. neoformans reads across various size ranges from eukaryote enriched sequenced pools.

Supplemental Table 1. Microbial members of a synthetic community. Species used in building a synthetic community is shown along with the primers and annealing conditions for qPCR.

Organism	Taxonomy	Gene Target	Primers (5' – 3')	Annealing Temp. C
Candida albicans	Fungi, Ascomycota	Protein Arginine Methyltransferase (*HMT1*)	1) TTG ACA AGC CCA AGG AAA TC<br>2) AAC CCG CCT TCA ACA AGA TA	57
Cryptococcus neoformans	Fungi, Basidiomycota	Transcription Initiation Factor (CNAG_02536)	1) GCA CGT AAG TCG CCG CTT TG<br>2) CTG TCC GCG CGG GAA TAA GT	57
Saccharomyces cerevisiae	Fungi, Ascomycota	Chromatin Silencing Protein (*SIR1*)	1) CGC AAG AAA CCG AAG GCA CG<br>2) ATG CTT GCA CTG GGC TCC TC	56
Leishmania major	Eukaryota, Euglenozoa	Nucleoside Diphosphate Kinase (ndpk)	1) TCG AGC GCA AGG GCT ACA AG<br>2) GCC GCT CTT CAC CAC GTT CT	57
Trypanosoma brucei	Eukaryota, Euglenozoa	Triose Phosphate Isomerase (Tb11.02.3210)	1) GAC GAA GGA GCG TCT TTC AC<br>2) ACC TTG TCC GCA ACA ATC TC	57
Clostridium difficile (toxigenic)	Bacteria, Firmicute	Metalloendopeptidase (CD630-06950)	1) TGA AGA AGC CTT GTC AAG ACC AAA<br>2) TCC TTG CTT ACT TGC TAT TTC AGT TCT	57
Limosilactobacillus reuteri (*Lactobacillus reuteri*)	Bacteria, Firmicute	**α**-Ketoacid Dehydrogenase **β** subunit (LMB90_RS03670)	1) ACG GTG GTG TTT TCC GTG CT<br>2) CGC ATC CGT GAC ATT TGG GC	57
Escherichia coli	Bacteria, Proteobacteria	L-cysteine Desulfurase (sufS)	1) GAC GCC GAG GCC GAG TTT TA<br>2) CCC CAG CTA TTG GCG ACC AG	57
Salmonella typhimurium	Bacteria, Proteobacteria	Transcriptional Regulator (hilA)	1) GCG ACG CGG AAG TTA ACG AAG A<br>2) GCA GAC TCT CGG ATT GAA CCT GAT C	57
Staphylococcus aureus (MRSA)	Bacteria, Firmicute	Staphylocoagulase (coa)	1) CGG AGC TCG TCC GAC ATA CA<br>2) TTG GGC GAG CGC CAT ATG AT	56

Supplemental Table 2. Sample preparation and post bioinformatic quality control. Each fecal (sample 21 and 25) was extracted using the optimized bead beating protocol (B), bead beating plus DpnI digestion (BE), the Qiagen Powersoil Extraction kit (Q), and FACS enrichment with bead beating protocol for Gate 1(G1) or Gate 2 (G2)

Sample	FACS event numbers	DNA concentration (ng/uL)	Raw reads	Post Trimmomatic	Adapter content	Human genomic removal reads	Percent human	Remaining Reads	Microbial eukaryote reads	*Cryptococcus* recovered reads
21B	n/a	24.1	13,307,080	11,605,196	30,453	6388	0.06%	11,598,808	3087	38,028
21BE	n/a	3.05	15,308,606	12,022,909	215,908	9547	0.08%	12,013,362	2393	33,054
21Q	n/a	5.0	11,799,156	8,443,161	74,315	4915	0.06%	8,438,246	7318	29,301
21G1	141,233	0.230	17,779,336	12,916,469	2,991,427	592,815	2.27%	12,323,654	69,174	253,328
21G2	146,929	To low	35,365,474	11,268,039	17,381,756	209,765	1.86%	11,058,274	27,990	248,620
25B	n/a	17.0	13,865,490	10,204,075	152,510	66,970	0.66%	10,137,105	3315	21,050
25BE	n/a	2.64	11,315,127	8,164,222	420,243	56,725	0.69%	8,107,497	3283	15,881
25Q	n/a	56.0	11,956,097	8,490,339	365,802	44,608	0.53%	8,445,731	3304	28,995
25G1	288,472	To low	37,436,007	2,810,230	29,887,858	409,941	14.49%	2,400,289	46,306	154,775
25G2	257,976	To low	35,463,511	2,953,462	17,179,387	606,047	20.52%	2,347,415	28,525	237,745

Supplemental Table 3. Comparison of fungal reads between the Human Microbiome Project and this study.

Species	HMP Samples n = 472	HMP Reads (27,091,491,028)	FACS Reads n = 2 (15,285,573)	Bead Beating Reads n = 2 (11,792,200)	Bead Beating + Enrichment Reads n = 2 (10,845,857)	Qiagen Reads n = 2 (9,615,294)
Malassezia restricta	191	5,829	4,461	84	56	62
Saccharomyces cerevisiae	198	6,205	13,814	425	171	1,776
Malassezia globosa	168	2,373	1,297	83	51	52
Cyberlindnera jadinii	92	88,922	22	0	0	0
Saccharomyces pastorianus	84	307	8,388	238	95	937
Candida albicans	55	2,426	34,132	64	82	90
Debaryomyces hansenii	32	278	36	0	0	0
Malassezia sympodialis	28	92	1,333	66	54	46
Alternaria alternata	24	81	224	0	0	0
Candida parapsilosis	25	158	1,200	62	28	74

Supplemental Methods section for eukaryome enrichment and DNA extraction

Note: A visual companion to this workflow is provided in the figure attached to this protocol.

FACS Enrichment:

1. Determine the specific population(s) of interest and optimize gating parameters using forward scatter and side scatter. Adjust the voltages and thresholds to optimize visualization and collection of your population(s) of interest.

NOTE: For this manuscript, the gates were built around *Cryptococcus neoformans* and *Staphylococcus aureus*.

2. Weigh 200 mg of feces and add 1 mL of 0.9% saline solution. Vortex the sample for 10 seconds at the highest speed. Centrifuge sample for 3 minutes at a speed of 300 xg. Gently remove the supernatant and save in 15 mL conical tube.
3. Repeat this step 2 three more times. Place 15 mL conical on ice once the fecal sample washing is complete.

Supplemen

NOTE: For every 100 mg of stool, add 500 μL of 0.9% saline solution.

NOTE: By the fourth wash, the supernatant should be only slightly cloudy or translucent.

4. Prepare controls for FACS sorting.
5. Sort cells according to the pre-determined gating strategies using a Fluorescent Activated Cell Sorter (Example: Aria III)
6. After FACS run, centrifuge samples for 30 minutes at 3000 rpm. Remove supernatant, resuspend in 500 uL of 1x PBS and move to the extraction protocol.

Extraction protocol for unprocessed feces:

1. Weigh 0.6 g of 500 nm glass beads and add to bead bashing tubes. Make one for each sample extracting.
2. Remove between 250 mg of stool sample and add 500 uL of TENT buffer to the mixture. Add mixture to a bead bashing tube and bead beat the mixture for 1 minute on 2 minutes off for 5 times.
3. Once bead beating is complete, add 300 µL of Phenol:Chloroform:Isoamly (25:24:1) Gently shake the tubes until the mixture is cloudy and centrifuge for 10 minutes at 12000 rpm at 4°C.

NOTE: Keep phenol:chloroform:isoamly and chloroform:isoamyl at 4°C before use.

4. Gently remove aqueous layer (top layer) without disturbing the middle protein-lipid layer, to a fresh chloroform resistant tube.
5. Add equal volumes of chloroform:isoamyl (24:1) to the aqueous layer. Gently shake the tube to homogenize the mixture, and then centrifuge for 10 minutes at 12000 rpm at 4°C.
6. Gently remove aqueous material (top layer) and add to a 15.0 mL conical. Add 3x volume of ice cold 95% ethanol and 1/10th volume of 3M Sodium acetate to the mixture. Gently invert to mix and then incubate at -20°C for at least 1 hour.
7. After the -20°C incubation, centrifuge the tube for 20 minutes at 12000 rpm at 4°C.

8. Gently remove 95% EtOH and gently wash the DNA pellet with 70% ethanol twice without removing DNA pellet from the centrifuge tube wall. Remove all traces of ethanol and allow DNA pellet to air dry (roughly 5 minutes).
9. Resuspend the DNA pellet in 50 uL of water.
10. For a final purification step, add 1.5x concentration of magnetic beads and incubate for 10 minutes to allow the DNA to attach to the beads.
11. Let mixture settle and then place tubes on magnetic plate and allow the tube to incubate for 3 minutes. After incubation, **remove supernatant** from the beads while still on magnetic plate. **KEEP BEADS**
12. Without removing the tubes from the magnetic rack, wash the beads with 200 uL of 80% ethanol twice.
13. Remove all traces of ethanol while on the magnetic rack and air dry for 5 minutes.
14. Vigorously add 50 uL of water to pellets and allow DNA to return to solution and incubate for 10 minutes. Added tube back to the magnetic rack and incubate for 3 minutes.
15. Remove aqueous material and place in new tube. This is your purified DNA. **KEEP AQUEOUS MATERIAL AND THROW AWAY BEADS**

Extraction protocol for FACS processed feces samples:

1. Weigh 0.6 g of 500 nm glass beads and add to bead bashing tubes. Make one for each sample extracting.
2. Add 500 μL of sorted cell suspension and 500 μL of TENT buffer. Add mixture to a bead bashing tube and bead beat the mixture for 1 minute on 2 minutes off for 5 times.
3. Once bead beating is complete, add 300 µL of phenol:chloroform:isoamly (25:24:1) Gently shake the tubes until the mixture is cloudy and centrifuge for 10 minutes at 12000 rpm at 4°C.
4. Gently remove aqueous layer (top layer) without disturbing the middle protein-lipid layer, to a fresh chloroform resistant tube.
5. Add equal volumes of chlorform:isoamyl (24:1) to the aqueous layer. Gently shake the tube to homogenize the mixture, and then centrifuge for 10 minutes at 12000 rpm at 4°C.
6. Gently remove aqueous material (top layer) and add to a 15.0 mL conical. Add 3x volume of ice cold 95% ethanol and 1/10th volume of 3M Sodium acetate to the mixture. Gently invert to mix and then incubate at -20°C for at least 1 hour.
7. After the -20°C incubation, centrifuge the tube for 20 minutes at 12000 rpm at 4°C.
8. Gently remove 95% EtOH and gently wash the DNA pellet with 70% ethanol twice without removing DNA pellet from the centrifuge tube wall. Remove all traces of ethanol and allow DNA pellet to air dry (roughly 5 minutes).
9. Resuspend DNA pellet with 50 μL of water. This is the purified DNA.

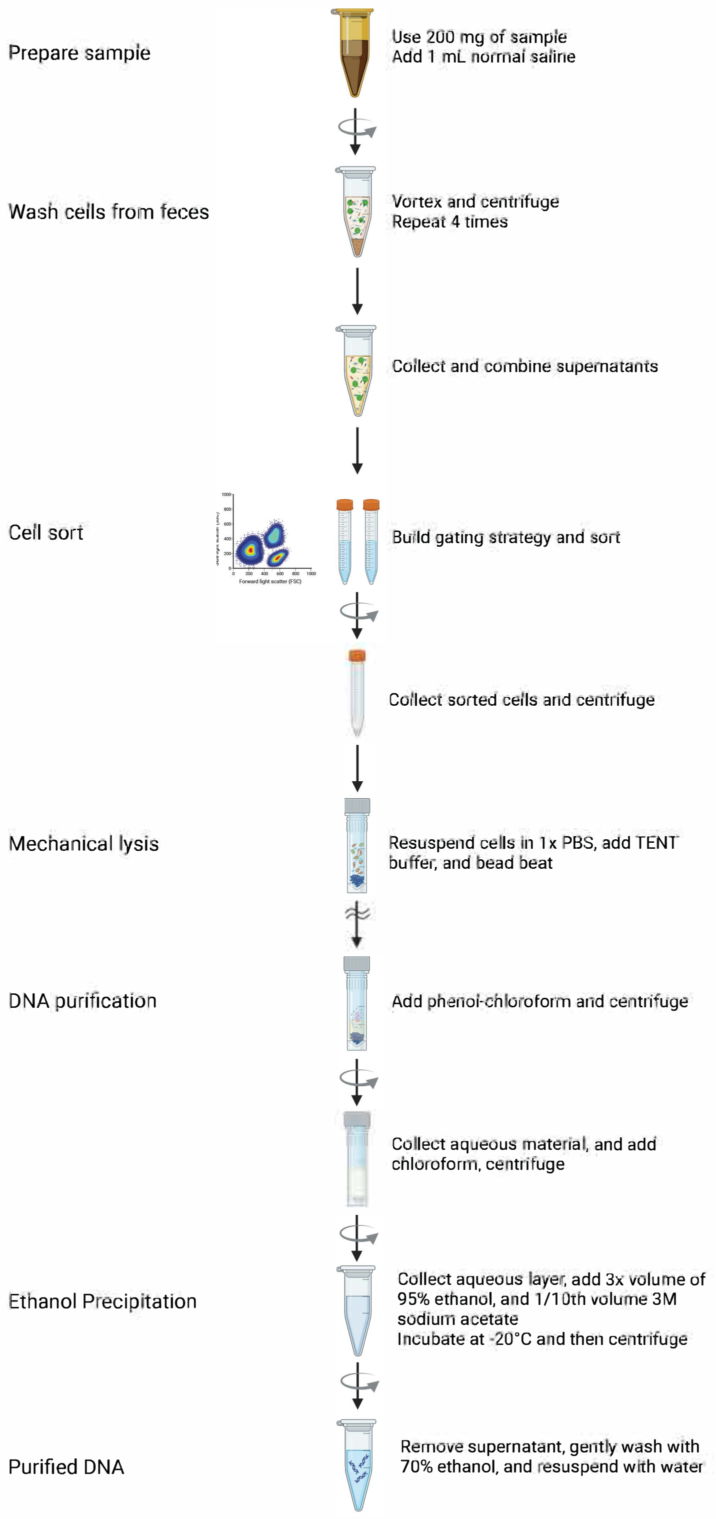

Supplemental Figure 10. Workflow for eukaryotic enrichment and metagenomic sequencing. A cartoon outlines the steps taken for enrichment of eukaryotes from stool samples followed by DNA isolation for metagenomic sequencing.
